# Functional capacities of microbial communities to carry out large scale geochemical processes are maintained during ex situ anaerobic incubation

**DOI:** 10.1371/journal.pone.0245857

**Published:** 2021-02-25

**Authors:** R. M. Wilson, A. A. Zayed, K. B. Crossen, B. Woodcroft, M. M. Tfaily, J. Emerson, N. Raab, S. B. Hodgkins, B. Verbeke, G. Tyson, P. Crill, S. Saleska, J. P. Chanton, V. I. Rich, IsoGenie Project Coordinators, IsoGenie Project Field Team

**Affiliations:** 1 Department of Earth Ocean and Atmospheric Sciences, Florida State University, Tallahassee, FL, United States of America; 2 Department of Microbiology, The Ohio State University, Columbus, OH, United States of America; 3 Australian Center for Ecogenomics, University of Queensland, Brisbane, Australia; 4 Department of Ecology and Evolutionary Biology, University of Arizona, Tucson, AZ, United States of America; 5 Department of Plant Pathology, University of California, Davis, CA, United States of America; 6 Stockholm University, Stockholm, Sweden; Tsinghua University, CHINA

## Abstract

Mechanisms controlling CO_2_ and CH_4_ production in wetlands are central to understanding carbon cycling and greenhouse gas exchange. However, the volatility of these respiration products complicates quantifying their rates of production in the field. Attempts to circumvent the challenges through closed system incubations, from which gases cannot escape, have been used to investigate bulk *in situ* geochemistry. Efforts towards mapping mechanistic linkages between geochemistry and microbiology have raised concern regarding sampling and incubation-induced perturbations. Microorganisms are impacted by oxygen exposure, increased temperatures and accumulation of metabolic products during handling, storage, and incubation. We probed the extent of these perturbations, and their influence on incubation results, using high-resolution geochemical and microbial gene-based community profiling of anaerobically incubated material from three wetland habitats across a permafrost peatland. We compared the original field samples to the material anaerobically incubated over 50 days. Bulk geochemistry and phylum-level microbiota in incubations largely reflected field observations, but divergence between field and incubations occurred in both geochemistry and lineage-level microbial composition when examined at closer resolution. Despite the changes in representative lineages over time, inferred metabolic function with regards to carbon cycling largely reproduced field results suggesting functional consistency. Habitat differences among the source materials remained the largest driver of variation in geochemical and microbial differences among the samples in both incubations and field results. While incubations may have limited usefulness for identifying specific mechanisms, they remain a viable tool for probing bulk-scale questions related to anaerobic C cycling, including CO_2_ and CH_4_ dynamics.

## 1. Introduction

Wetlands contain enormous reservoirs of carbon (C) [1] and are a significant ecosystem in northern and boreal regions based on large areal extent [2]. Wetlands produce up to a quarter of the total global methane (CH_4_) flux [2], a greenhouse gas second only to CO_2_ in terms of warming potential. Further climatic warming, even without accounting for the complex effects of changing hydrology, is expected to increase C emissions from wetlands, especially peatlands [3], creating a positive feedback with climate [2,4]. Despite the importance of this process, uncertainties regarding C emission flux estimates from wetlands remain large [1,5], in part because the mechanistic controls on CO_2_ and CH_4_ production, and the microbial processes that mediate them, remain poorly understood.

*In situ* environmental controls on C emission rates tend to be numerous, complex, and interdependent, such that they can be difficult to quantify except under controlled experimental settings [3]. Incubations are a commonly employed method for studying climatically relevant biogeochemical processes in natural ecosystems, particularly peatlands [3,6–10] as they offer several critical advantages over *in situ* field observations, including control and isolation of variables that fluctuate in complicated and interdependent ways in the field (e.g. temperature, water table depth, etc.), the opportunity for direct manipulation, and the improved potential for detailed measurements. For example, closed incubations are hydrologically isolated and thereby eliminate gas loss through difficult-to-capture pathways like aerenchymous transport or horizontal flow, simplifying interpretation of CO_2_ and CH_4_ dynamics [9,11]. Measurements can be repeated at high resolution over longer timescales in incubations, allowing exploration of kinetic effects [12], mechanisms of C cycling/processing, and the relative impacts of controls on C gas production such as organic matter quality, C and N mineralization potential and C partitioning [3].

However, *ex situ* incubations inherently impose changes relative to field conditions, both through the disturbance of collection and storage, which can, among other effects, introduce oxygen into previously anaerobic or microaerobic materials, and their intrinsic nature as closed systems which can cut off the supply of critical substrates or create conditions under which toxic byproducts accumulate to inhibitory concentrations. Additionally, microbial community disruption during sampling and storage can alter ecosystem processes [13], and in particular, handling of peatland samples for incubation setup can alter the methanogenic microbial communities [14], likely due to the oxygen-sensitivity of key methanogenesis enzymes [15]. This could contribute to the frequently observed delay in CH_4_ production in anoxic incubations [16], though the prerequisite depletion of residual molecular O_2_ or alternative terminal electron acceptors may also play a role [7]. The closed nature of laboratory incubations eliminates the spatial connectivity and dynamism of the field, which may be important for controlling ecosystem outputs of interest such as carbon gas emissions. For example, substrates may be resupplied through aboveground-belowground connections, toxic metabolites may be removed through hydrologic flow, or shifting water tables may create aerobic microsites sustaining methanotrophy during periods of inundation. In incubations from aquatic systems, these closed-system impacts collectively create the ‘bottle effect’, whereby observed microbial community changes may be due to confinement rather than the intended experimental manipulation [17]. Described bottle effects in aquatic systems include a decreased autotroph:heterotroph biomass ratio [18], and an increase in ***γ****-Proteobacteria* [19], both potentially skewing observed processes and communities.

In spite of these caveats, numerous studies in varied peat sites across the world continue to use incubations to study peatland processes and the results of these studies suggest that closed-system anaerobic incubations largely reproduce *in situ* geochemical processes, including CO_2_ and CH_4_ production and dynamics, and organic matter transformations based on bulk characterizations by spectroscopic methods (e.g Fourier Transform Infrared Spectroscopy, FT-IR, Excitation Emission Matrix Spectroscopy, EEMS, Ultraviolet-Visible spectroscopy UV-Vis) [3,6,8,9,11,20]. This suggests that closed incubation microbial communities may, at least functionally, represent field communities. Such functional duplication could be achieved if the microbial response to disturbance is resistance (composition and function do not change), resilience (community composition returns to original composition after temporary alteration), or permanently altered community composition that retains functional redundancy [13].

Generally, there can be a high degree of overlap in substrate utilization and/or functional redundancy within microbial communities. Such functional redundancy may allow communities to regain their *in situ* functionality following disturbance [21–23]. Soil heterogeneity may preserve micro-pockets of functional guilds in spite of transient O_2_ infiltration, drying, or temperature fluctuations during sampling and storage [24,25]. Previous peat incubations have been used to assess potential for differences in greenhouse gas production dynamics [9] and various microbial production pathways (e.g. methanogenesis, sulfate and iron reduction, denitrification, fermentation) from different habitats or under different conditions, or environmental controls over these pathways (e.g. pH) [7,10,26]; however, to our knowledge, none have considered disturbances to the microbial communities imposed by sample collection and storage, and evaluated the similarity to field communities or considered both high resolution geochemical changes by advanced analytical techniques (Fourier Transform Ion Cyclotron Resonance Mass Spectrometry, FTICR-MS) in concert with microbiological changes.

In this study, we used tandem microbial and geochemical analyses of incubated peats from three wetland habitats to understand the effect of perturbation during incubation on interpretation of results. Geochemistry and microbiology were characterized at time of collection, after storage, and periodically throughout the incubations, to quantify changes in dissolved organic matter, solid organic carbon and microbial community over time. We hypothesized that the geochemical characteristics would shift during storage and incubation consistent with decomposition by a microbial community of decreased diversity. We explored observed differences to understand how the changes observed in the microbial community were likely to influence the ultimate geochemical understanding of these sites when studied using *ex situ* methods.

## 2. Materials and methods

### 2.1 Ethics statement

Under the IsoGenie Project, our team maintains field permits with the Abisko Scientific Research Station and its parent organization, the Swedish Polar Research Secretariat. For importation of this material into the United States, peat is considered exempt from soil import regulations under USDA Circular Q-330.300–1. The importing lab also held a soil import permit for soils from this field site, USDA Soil Import Permit # P330-14-00283.

### 2.2 Sample collection

Stordalen Mire is a thawing permafrost peatland in the discontinuous permafrost zone of subarctic Sweden. Over the past several decades, permafrost thaw has caused flooding and plant community shifts, creating a mosaic of habitats spanning a gradient of permafrost degradation [27–29]. The sites in our study represent the inundated and partially-inundated stages of the thaw gradient and include a recently-thawed thermokarst sinkhole (collapsed palsa; referred to as “PHB”, i.e. palsa hole-big), a partially thawed *Sphagnum*-dominated bog, and a fully thawed *Eriophorum*-dominated fen. Under the IsoGenie Project, our team maintains field permits with the Abisko Scientific Research Station and its parent organization, the Swedish Polar Research Secretariat to collect samples from Stordalen Mire.

Porewater and peat cores were collected from three habitat types spanning the thaw gradient: collapsed palsa (PHB, N 68 21.1800, E 19 02.8410), bog (N 68 21.1973, E 19 02.8537), and fen (N 68 21.1992, E 19 02.8063) during the peak of the growing season (July 16 2014 for bog, July 17 2014 for fen, and July 22 2014 for PHB). Peat was collected from the bog site using a push-core and from the collapsed palsa and fen sites using an Eijelkamp corer (Eijelkamp, The Netherlands). Porewater was collected using stainless steel piezometers that were pushed into the peat to the desired depth, then water was extracted using an airtight syringe. Porewater was filtered to 0.7 μm in the field and then one aliquot was frozen at -20°C for DOC analysis while a second aliquot was preserved with 10% phosphoric acid for CH_4_ and dissolved inorganic carbon (DIC) analysis. Aliquots of peat from each core taken from 8–18 cm and 28–38 cm below the surface were preserved for geochemical analysis by freezing at -20°C. A second aliquot of peat from each of these depths in the bog and fen sites was preserved for microbial community analysis by saturating in LifeGuard and freezing at -80°C. Approximately 1 kg of peat was removed from the walls of each of the core holes at 8–18 cm and 28–38 cm below the surface for laboratory incubation experiments. Peat for incubations was stored following the approach outlined in Hodgkins et al., [8,30] i.e. in plastic bags at 2°C.

### 2.3 Incubation experiments

Anaerobic incubation experiments were conducted after approximately 4 months of peat storage at 2°C. No attempt was made to maintain the peat under anaerobic conditions during this storage time. Thus, peat was pre-incubated under anaerobic conditions for 25 days before the initiation of the experiment to reduce compounds that had been oxidized during storage and to allow microbial communities to re-equilibrate. Twenty-five days has been shown to be sufficient for exhausting oxidized electron acceptors in other similar peat incubation experiments [31] and 10-25-day pre-incubations are frequently used in anaerobic incubation studies [8,9,32,33]. After the 4-month storage, but before pre-incubation, the 1-kg samples of peat from each site/depth were gently homogenized by hand to minimize mechanical breakage of fibers and subsequent leaching. An aliquot was taken from each of the homogenized peat samples for geochemical and microbial analyses. These samples are referred to as the “setup” samples in the remainder of the text and represent changes after 4 months of cold storage under aerobic conditions. The remaining homogenized peat was then apportioned into 125 mL glass serum vials (6–8 replicates per site/depth combination, 40 g per vial). To each vial, 40 mL of distilled water was added and the vials were capped with a septum and aluminum crimp to make them gas-tight. The vials were then flushed with N_2_ for 5 minutes, shaken for two minutes and this was repeated until the CO_2_ concentration in the headspace was <0.5% as measured by gas chromatography. After flushing, the vials were placed in a dark location maintained at 18°C throughout the 25-day pre-incubation period. Field temperature at the site averaged <15°C [30]; however, incubations were conducted at 18°C to ensure CO_2_ and CH_4_ production rates were tractable for the timescale of this experiment as is commonly done in previous incubation experiments [7,8,30]. At the end of the 25 days, 2 replicates from each habitat/depth incubation set were sacrificed for microbial analyses. Porewater from the incubations was extracted by filtration and used for spectroscopic (UV-Vis and fluorescence) and spectrometric (Fourier transform ion cyclotron resonance mass spectrometry) analyses as described below. The remaining peat was preserved for solid-phase geochemical analyses. These samples were referred to as the day 0 samples throughout the remainder of the text. The remaining incubation vials were then flushed with N_2_ and shaken as before until CO_2_ headspace concentrations were <0.5% to remove any oxygen that may have been evolved during the pre-incubation period. These steps were thought to be sufficient to produce highly anaerobic conditions within the vials, conducive to methanogenesis [30]. The vials were not flushed again during the remainder of the experiment.

CO_2_ and CH_4_ concentrations were monitored by headspace analysis approximately every 3–5 days. Initially, the concentrations were measured by gas chromatograph, but once concentrations were high enough (>1000 ppm) concentrations and stable carbon isotopes were measured simultaneously by isotope ratio mass spectrometry (see next section). Incubations were allowed to proceed for 50 days; after 25 days 2 replicates were sacrificed for microbial analysis. At the end of 50 days, two additional replicates were sacrificed for geochemical and microbial analysis as described below. These were referred to as the day 25 and day 50 samples, respectively. Another set of incubations were allowed to proceed for a total of 100 days, during which gas production (CO and CH_4_) was monitored, but the detailed geochemistry and microbiology was not.

### 2.4 Geochemical analyses

Porewater dissolved CH_4_ and DIC concentrations and isotopes for field and incubation samples were obtained concurrently on a Delta MAT Finnigan Isotope Ratio Mass Spectrometer by direct injection after headspace equilibration. DOC concentration was measured using a Shimadzu TOC-VCP analyzer. Concentrations were calculated relative to a prepared standard curve analyzed concurrently with field samples. UV/Vis absorbance of the porewater samples, both field and incubation, between 240 and 800 nm were measured on a Cary Varian 100 dual beam spectrometer, blank-corrected against milli-Q distilled water. The specific absorbance at 254 nm (SUVA_254_ L mgC^-1^ m^-1^) was calculated from the absorption coefficient at 254 nm divided by the DOC concentration (in mg L^-1^). This measure has been shown in other peatlands to correspond to aromatic content of the DOM [34]. The spectral slope ratio for each spectrum was also calculated as the ratio of first derivative of the log-transformed spectra over the range 275–295 nm to the first derivative over 350 to 400 nm. This measurement of spectral slope has been shown to account for the largest amount of variation in the optical properties of environmental samples [35] and is thought to be inversely related to the average molecular weight of optical compounds present in a sample [34,36]. Fluorescence Excitation-Emission Matrix Spectroscopic (EEMS) measurements were made on a SPEX Fluoromax-4 spectrometer. All samples were diluted to a common absorbance of 0.02AU at 350 nm to reduce inner-filter effects. Emission was recorded between 290 to 600 nm (in 2 nm increments) at excitation wavelengths scanned from 240 to 500 nm (at 5 nm increments). Data were analyzed in MATLAB2015a using FL toolbox 1.91 to correct for dilution, remove Rayleigh and Raman scattering peaks [37], normalize to Raman intensity [34,38], and convert to quinine sulfate equivalents (QSE) in ppb [39]. Areas of commonly occurring DOM peaks as defined by Coble [40] were integrated for comparison among samples.

Porewater samples were also analyzed using direct-inject high resolution mass spectrometry (12T, FTICRMS) at the Environmental Molecular Sciences Laboratory (EMSL) in Richland, Washington, USA to identify more than 10,000 compounds comprising the dissolved organic matter (DOM). The compound identification algorithm (CIA) of Kujawinski and Behn [41], as adapted by Minor et al. [42] was used to assign molecular formula to identified masses.

Solid peat from both the field and incubations was freeze-dried and ground to a fine powder for Fourier transform Infrared analyses (FTIR). % Transmittance was measured from 4000–650 cm^−1^ on a PerkinElmer Spectrum 100 FTIR spectrometer with a zinc selenide/diamond attenuated total reflectance (ATR) accessory. Resulting spectra were baseline-corrected and ATR-corrected with the instrument software, then converted to absorbance. Individual peaks were separately baseline-corrected, and peak areas above the baselines then normalized to the area of the whole spectrum, according the procedure of Hodgkins et al., [43]. Aromatics, carbohydrates, organic acids, and lipid contributions to the spectra were calculated as the baseline corrected, normalized peak areas [43].

### 2.5 16S-based cell abundances and microbial community profiling

#### 2.5.1 Nucleic acid extraction

For replicate peat samples from the field, incubation setup, and days 0, 25, and 50 of incubation, DNA extractions were performed as described by Mondav et al., [44] and Woodcroft et al., [45]. Briefly, total nucleic acids were extracted from ~2 g peat sample using the PowerMax Total Nucleic Acid extraction kit (MoBio), retaining the LifeGuard preservation solution during lysis. DNA was purified by RNaseA digestion, phenol-chloroform-isoamyl alcohol purified and ethanol precipitated. Approximately 15 ng DNA from each sample was used as a template in PCR reactions. Libraries for low concentration DNA samples were created using 1 ng of DNA with the Nextera XT DNA Sample Preparation Kit.

#### 2.5.2 Quantitative PCR

As a proxy for total abundance of microbial cells, quantitative polymerase chain reaction (qPCR) of the 16S rRNA gene was conducted for each community. Each reaction contained 5 μl of 2X SYBR Green PCR Master Mix (Applied Biosystems, Carlsbad, CA, USA), 4μl of template DNA, and 1μl of primer mix. Bacterial and archaeal 16S rRNA genes were amplified using the primer set 1406F/1525R (0.4 μM, F—GYACWCACCGCCCGT and R—AAGGAGGTGWTCCARCC). For inhibition control samples, the rpsL primer pair (0.2 μM, F—GTAAAGTATGCCGTGTTCGT and R—AGCCTGCTTACGGTCTTTA) was used to amplify *Escherichia coli* DH10B only. Each sample and standard were run in triplicate, with three dilutions (1/100, 1/500, and 1/1000) and an inhibition control (1/100 dilution of E. coli DH10B genomic DNA spiked into a 1/100 dilution of the sample). *E*. *coli* DH10B genomic DNA dilutions of 10^−2^, 10^−3^, 10^−4^, 10^−5^ and 10–6 of the 20 ng/μl stock solution were used for the standards. The ViiA7 Real-Time PCR System (Applied Biosystems, Carlsbad, CA, USA) was used for the qPCRs, with the following cycling conditions: 10 min at 95°C, 40 cycles of [15 s at 95°C, then 20 s at 55°C, then 30 s at 72°C]. To produce a melt curve, a cycle of 2 min at 95°C was run, and then a final cycle of 15 s at 60°C. The cycle threshold (Ct) values were recorded and analyzed using ViiA7 v1.2 software, and 16S rRNA gene copy numbers were calculated for each sample. These calculations accounted for the genome size (4,686,137 bp) and 16S rRNA gene copy number (7) of the standard. To account for variance in genome copy number of the 16S gene between different microbial lineages for more accurate cell numbers, the resulting qPCR counts were processed using CopyRighter [46].

#### 2.5.3 16S PCR amplification and amplicon sequencing

The 16S rRNA gene encompassing the V6 to V8 regions was targeted using the 926F (5’-AAACTYAAAKGAATTGRCGG-3’) and 1392R (5’-ACGGGCGGTGWGTRC-3’) primers [47] modified to contain Illumina specific adapter sequence (926F:5’TCGTCGGCAGCGTCAGATGTGTATAAGAGACAGAAACTYAAAKGAATTGRCGG3’ and 1392wR:5’GTCTCGTGGGCTCGGGTCTCGTGGGCTCGGAGATGTGTATAAGAGACAGACGGGCGGTGWGTRC3’). The universal primer pair Univ_SSU_926F-1392wR amplifies the small subunit (SSU) ribosomal RNA of prokaryotes (16S), specifically the V6, V7 and V8 regions. In *E*. *coli*, it amplifies the 926–1392 region of the 16S gene.

Preparation of the 16S library was performed as described, using the workflow outlined by Illumina (#15044223 Rev.B). In the 1st stage, PCR products of ~466bp were amplified according to the specified workflow with an alteration in polymerase used to substitute Q5 Hot Start High-Fidelity 2X Master Mix (New England Biolabs) in standard PCR conditions. Resulting PCR amplicons were purified using Agencourt AMPure XP beads (Beckman Coulter). Purified DNA was indexed with unique 8bp barcodes using the Illumina Nextera XT 384 sample Index Kit A-D (Illumina FC-131-1002) in standard PCR conditions with Q5 Hot Start High-Fidelity 2X Master Mix. Indexed amplicons were pooled together in equimolar concentrations and sequenced on MiSeq Sequencing System (Illumina) using paired end sequencing with V3 300bp chemistry in the Australian Centre for Ecogenomics according to manufacturer’s protocol.

#### 2.5.4 iTag processing

16S amplicons were processed using the Australian Center for Ecogenomics (ACE) mitag pipeline (AMP). Amplicon sequences were processed by taking the forward read only, using Trimmomatic 0.32 with parameters "SLIDINGWINDOW:4:15 LEADING:10 HEADCROP:23 MINLEN:250" and then further processed using QIIME 1.8.0 pick_open_reference_otus.py script [48], assigning taxonomy using a combined GreenGenes 2013/05 [49] and the eukaryotic sequences from Silva 111 [50], using the 97% OTU clustered representative sequences from each set. Taxonomy was assigned with BLAST+ 2.3.30+ {blast}. OTUs where abundance filtered using the "—min_count_fraction = 0.0005" of the filter_otus_from_otu_table.py QIIME script. The first 20 bases of all fastq files were then trimmed to remove primer sequence, and quality trimmed to remove poor quality sequence using a sliding window of 4 bases with an average base quality above 15 using the software Trimmomatic. All reads were then hard trimmed to 250 bases, and any with less than 250 bases excluded. Fastq files were then converted to fasta files. Fasta files were processed using QIIME’s pick_open_reference_otus.py workflow with default parameters (97% similarity) and taxonomy assignment and alignment features suppressed. The resulting OTU table was filtered to remove any OTU with an overall abundance of less than 0.005%. Representative OTU sequences were then BLASTed against the reference database (Greengenes version 2013/05 for 16S).

#### 2.5.5 iTag analysis

Relative abundances of OTUs were determined by normalizing counts from the ACE mitag pipeline to the total number of sequences in each sample. Normalized OTU data was imported into Microsoft Excel and used to sum the relative abundances of microbial at taxonomic levels of phylum, class, and order (based on the Greengenes-assigned taxonomy), which were averaged between the two replicates for each time point and graphed. The R package vegan v2.4–3 [51] was used to calculate alpha diversity metrics (richness, Shannon index, and Pielou’s J). Richness values were rarefied to the sample with the lowest number of sequences. Weighted Fast Unifrac distances [52] using the normalized abundances were calculated between all samples using the R package phyloseq v1.20.0 [53] in RStudio. Bray Curtis dissimilarities of the relative abundances at higher taxonomic levels (phylum, class, and order), and the OTU level, were also calculated using vegan.

Principal coordinates analyses (PcoA) of the weighted Unifrac distances at the OTU level, and Bray Curtis dissimilarities of phylum, class and order relative abundances, were done with vegan, to investigate similarities among the microbial communities at various taxonomic levels (ranging from phylum to OTU). PcoA and alpha diversity measures were plotted using ggplot2 v2.2.1 [54]. Pairwise permutational analyses of variance with 9999 permutations of the weighted Unifrac distances, and Bray Curtis dissimilarities, were done in PRIMER v7 [55] using PERMANOVA+ add-on software [56]. Monte Carlo p-values for pairwise time point comparisons within each site/depth combination were reported.

#### 2.5.6 Prediction of microbial community functions

PICRUSt [57] from the package “themetagenomics” [58] in R was used to infer the functional profiles of the microbial communities from the copy number-corrected 16S rRNA data using the GreenGenes [49] v13.5 taxonomy. No further 16S rRNA copy number correction or sample normalization was carried out during the PICRUSt predictions (cn_normalize = FALSE,sample_normalize = FALSE). PICRUSt algorithm reconstructs the extended ancestral-state from the 16S information to predict gene families and to estimate metagenomes, with an average accuracy of 81% for soils collected from diverse geographic locations [57]. To account for uneven sequencing depth across samples, the predicted, KEGG-hierarchy-organized, metagenomes were then rarefied to 500 KEGG functions per metagenome using the “rrarefy” function of the package “vegan” [51] in R. Samples with less than 400 non-redundant KEGG functions (using the “specnumber” function of “vegan”) were excluded from downstream analyses to avoid clustering patterns due to under-sampling of the environment. The excluded samples are two shallow (S8670 & S8671) and one deep (S8673) setup collapsed palsa samples, one shallow (SA2281) and two deep (SA2280 & SA2282) field bog samples, and all the shallow (S8666 & S8667) and deep (S8668 & S8669) setup bog samples. Principal Coordinate analysis was conducted on a Bray-Curtis dissimilarity matrix of the predicted functional profiles after cube transformation. The Bray-Curtis dissimilarity matrix was calculated using the function “vegdist” with the method “bray”, while the ordination was conducted using the function “capscale” with no constraints (distance_matrix~-1); both functions are part of the package “vegan” in R. The ordination figure was then imported to Inkscape to draw the background polygons around the samples originating from the same habitats. The heatmap was constructed by first averaging the relative abundances of the replicates from the same habitat, depth, and timepoint then summing up all the relative abundances for the KEGG functions within the same KEGG module for the same habitat, depth, and timepoint. Only the “metabolic” modules in the KEGG hierarchy were considered in this analysis. Relative abundance values in the heatmap cells were scaled (4^th^ root-transformed) to better visually represent the low abundance modules in the color scale. All code is available via Zenodo (10.5281/zenodo.4081351).

#### 2.5.7 Metagenome sequencing

For 6 selected samples, from field, day 0, and day 50 for the bog and fen, metagenome sequencing was also performed using 100 ng of the extracted DNA in TruSeq (Illumina) library preparations (field samples) or TruSeq Nano (Illumina) library preparations (incubation samples), using an Illumina NextSeq500. The small sample size of these metagenomes precluded statistically meaningful comparisons of the overall community, so analyses focused on the methanogens.

#### 2.5.8 mcrA analysis from metagenomes

Due to the central role of methanogenesis in this study, we analyzed the McrA gene from the metagenomes to gain a complementary perspective on functional consistency of the methanogen community over the course of the incubations. McrA gene abundances were determined gene-centrically using GraftM 0.11.1 [59] with an McrA package (S1 Table), using default parameters except assigning taxonomy with the DIAMOND method [60].

## 3. Results and discussion

### 3.1 Geochemistry comparisons in incubations and field

#### 3.1.1 CO_2_ and CH_4_ production dynamics

**Fig 1:**
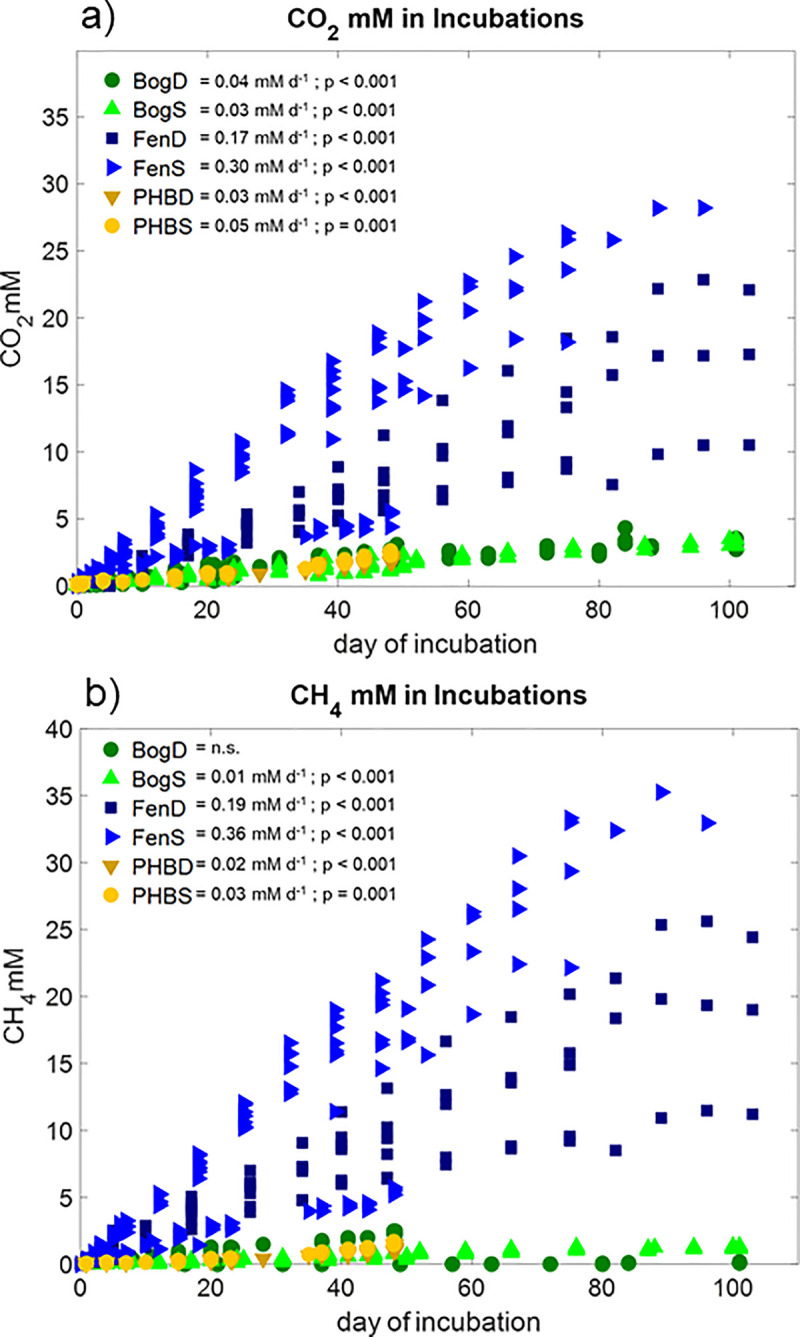
CO_2_ (panel a) and CH_4_ (panel b) production in the incubations. Linear regression analyses were used to calculate production rates in the incubations over time. The results for significant linear regressions are given in each panel.

Production rates of CO_2_ and CH_4_ in the incubations were nearly constant over time (Fig 1A and 1B), and generally increased from bog, to collapsed palsa, to fen. This trend in production rates among habitats reflects the trend in CO_2_ and CH_4_ emission rates observed in the field across these habitats, and likely results from the inhibition of microbial activity imposed by *Sphagnum* mosses in the bog, and the fueling of anaerobic decomposition in the fen by the vegetation’s more labile material [30]. Although we expect the magnitude of production rates in the incubations to be higher than field conditions (in part, because of the warmer temperatures used during the experiment) the preservation of relative production rates among habitats in the incubations suggests that the peat itself exerts a strong control on CO_2_ and CH_4_ production which is preserved in the incubations. That this control is conserved in the incubations suggests that the incubations may prove useful for probing the mechanisms underlying environmental controls on greenhouse gas production.

The CO_2_:CH_4_ ratio is an important measure of ecosystem greenhouse gas partitioning. Since CH_4_ is a much stronger greenhouse gas than CO_2_, this ratio reflects changes in climate forcing from changing decomposition pathways [61] and is an important parameter to consider when investigating peatland-climate feedbacks. We compared the CO_2_:CH_4_ ratios measured in the field to those measured in the incubations averaged over several different time points throughout the incubations (Fig 1). We found that the CO_2_:CH_4_ ratios in the incubations were not significantly different from the field at any time point during the incubations of deep collapsed palsa, shallow bog, or near the end of the incubations of shallow fen peats (Anova, p > 0.05). No incubations from the collapsed palsa were run for more than 50 d (due to lack of material collected in the second phase of the experiment). The CO_2_:CH_4_ ratio at the start of the fen incubations was highly variable compared to later in the incubations when it approached a 1:1 CO_2_:CH_4_ production ratio. It is expected that as decomposition advances, the system shifts towards more methanogenesis (i.e. [32,33,62]) and approaches this 1:1 ratio. In the shallow bog, the CO_2_:CH_4_ ratio at the start of the incubations was highly variable, as was the range observed in the field samples. Over time, the shallow bog incubations became less variable and approached a CO_2_:CH_4_ ratio ~2, which was not significantly different from the field and much higher than expected for a methanogenic system. This could suggest that some factor is limiting methanogenesis in the bog relative to the fen or that alternative electron acceptors are present in the bog that allow bog peat to sustain a high CO_2_:CH_4_ ratio.

The field CO_2_:CH_4_ ratio in the deep collapsed palsa was highly variable and did not differ significantly from the incubation ratios, although over time, the average CO_2_:CH_4_ declined somewhat. While high CO_2_:CH_4_ ratios may call into question the existence of methanogens at this site, previous work from our group has measured methanogens in peat collected from the collapsed palsa feature confirming that there are methanogens at this site [63]. The decline in CO_2_:CH_4_ ratios is consistent with increasing decomposition of the peat over time—expected in the incubations—leading to a more methanogenic system. The shallow collapsed palsa CO_2_:CH_4_ ratio was the highest of all the habitats in the field and much higher relative to the incubations. This could suggest that the material in the collapsed palsa, which has thawed more recently than the other habitats, contains fresher organic matter, and that the collapsed palsa becomes more methanogenic with increasing decomposition as those fresh substrates are used up during the incubations. Relatively higher CO_2_ production in the field could imply that conditions were more methanogenic in incubations than *in situ* due to the exhaustion of terminal electron acceptors (TEAs) during the pre-incubation period [64]. Additionally, the reaction rates for methanogenesis are generally more sensitive to temperature increase than the reaction rates for CO_2_ production [65,66], thus the higher temperature of the incubations relative to field conditions (18°C vs. average annual temperature 10°C; a difference necessary to achieve tractable gas production rates) may have stimulated a disproportionate increase in CH_4_ production rates [9].

**Fig 2:**
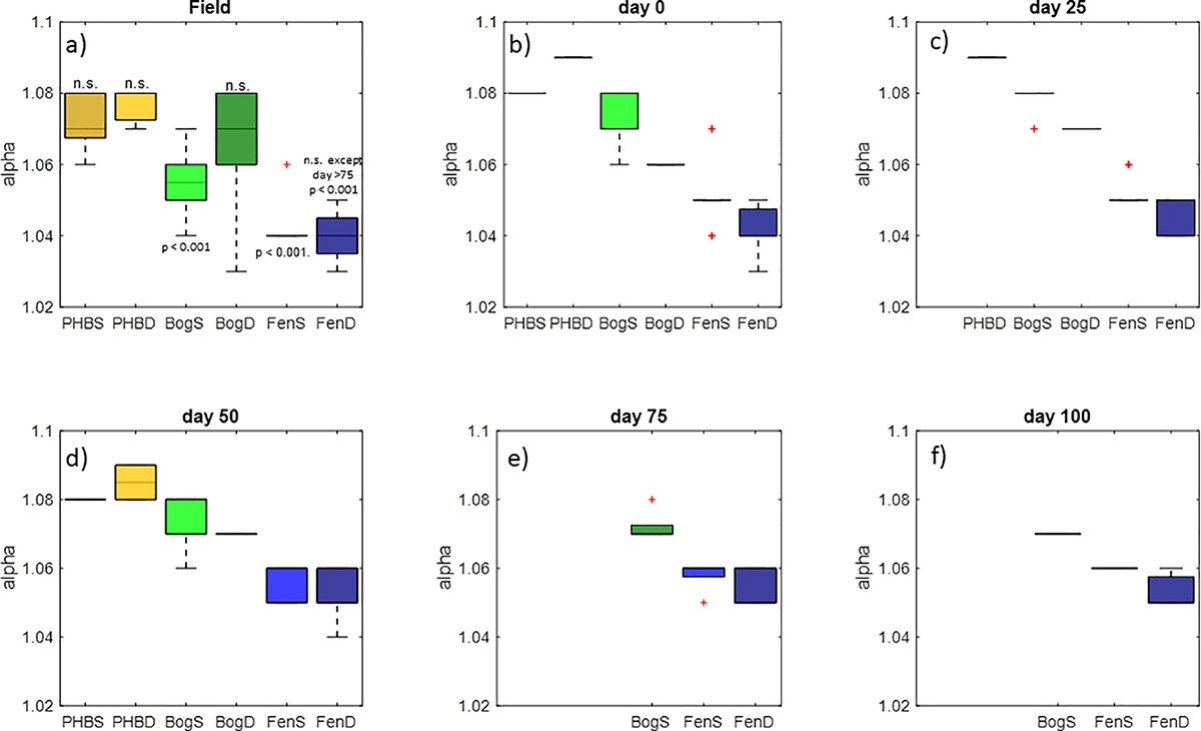
Alpha compared across habitats in the field (panel a) and and in the incubations averaged over 0–5 days (panel b), 20–30 days (panel c), 45–55 days (panel d), 70–80 days (panel e), and 90–100 days (panel f) throughout the incubation for each habitat. The first panel contains significance results of anova comparing the field and timepoints for each habitat. Neither depth of the collapsed palsas or BogD field were significantly different from any time point in the respective incubations. FenD was not significantly different until the end (day 75) of the incubations. BogS and FenS were the only sites that were significantly different from the incubations at all time points.

Another often-explored process in peatland ecology is the pathway by which methane is produced. Acetoclastic methane production (i.e. disproportionation of acetate into CO_2_ and CH_4_) and hydrogenotrophic methane production (reduction of CO_2_ to methane by hydrogen) are the two dominant pathways, although methylotrophic methanogenesis may also play a minor role in some peatlands [67]. To further investigate the processes underlying the observed carbon gas dynamics, we compared the carbon isotopic separation of CO_2_ and CH_4_ in the incubations to those from the field porewater (Fig 2). Because microorganisms preferentially consume the lighter isotope-bearing CO_2_ during hydrogenotrophic methanogenesis, calculating the isotopic difference between the CO_2_ and CH_4_ can give an approximate indication of the relative importance of hydrogenotrophy versus acetoclastic methanogenesis (which also fractionates, but to a lesser extent) [68]. This is typically done by calculating alphas ([δ^13^CO_2_+1000]/[δ^13^CH_4_+1000]), a larger alpha indicates hydrogenotrophic methanogenesis, while lower alphas are associated with acetoclasty. As previously reported by Hodgkins et al. [9] for Stordalen Mire, the collapsed palsa had the most hydrogenotrophic alphas (Fig 2), while methane production in the fen was strongly influenced by acetoclastic processes. There were no significant differences in alpha between the field and incubations in approximately half of the comparisons (Fig 2). Though, in general, alphas in the incubations were slightly higher (i.e. more hydrogenotrophic) compared to the field sites. This may have been a consequence of the more advanced state of decomposition in the incubations relative to the field which would tend to increase hydrogen availability making CO_2_ reduction more favorable. Nevertheless, although there were differences in magnitude between the field alphas and incubations for some comparisons, the general trend in alphas across the thaw gradient was preserved wherein collapsed palsa > bog > fen. Furthermore, the differences in alpha between the field and incubations, though significant, were small in magnitude and generally did not alter interpretations. The incubations correctly reflect the dominant hydrogenotrophic processes in the collapsed palsa, the mixture of hydrogenotrophic and acetoclastic methanogenesis in the bog, and the high proportion of acetoclastic methanogenesis in the fen. Collectively, the similarity in trends of alpha across habitats between the field and incubation samples, the similarity of trends in production rates relative to field emission rates and the similarity of CO_2_:CH_4_ ratios in the incubations compared to the field samples, supports the use of closed-system incubations for reproducibly studying anaerobic carbon cycling processes–and most specifically and climate-relevantly, CH_4_ production dynamics–in the field.

#### 3.1.2 Geochemistry of dissolved organic matter (DOM)

**Fig 3:**
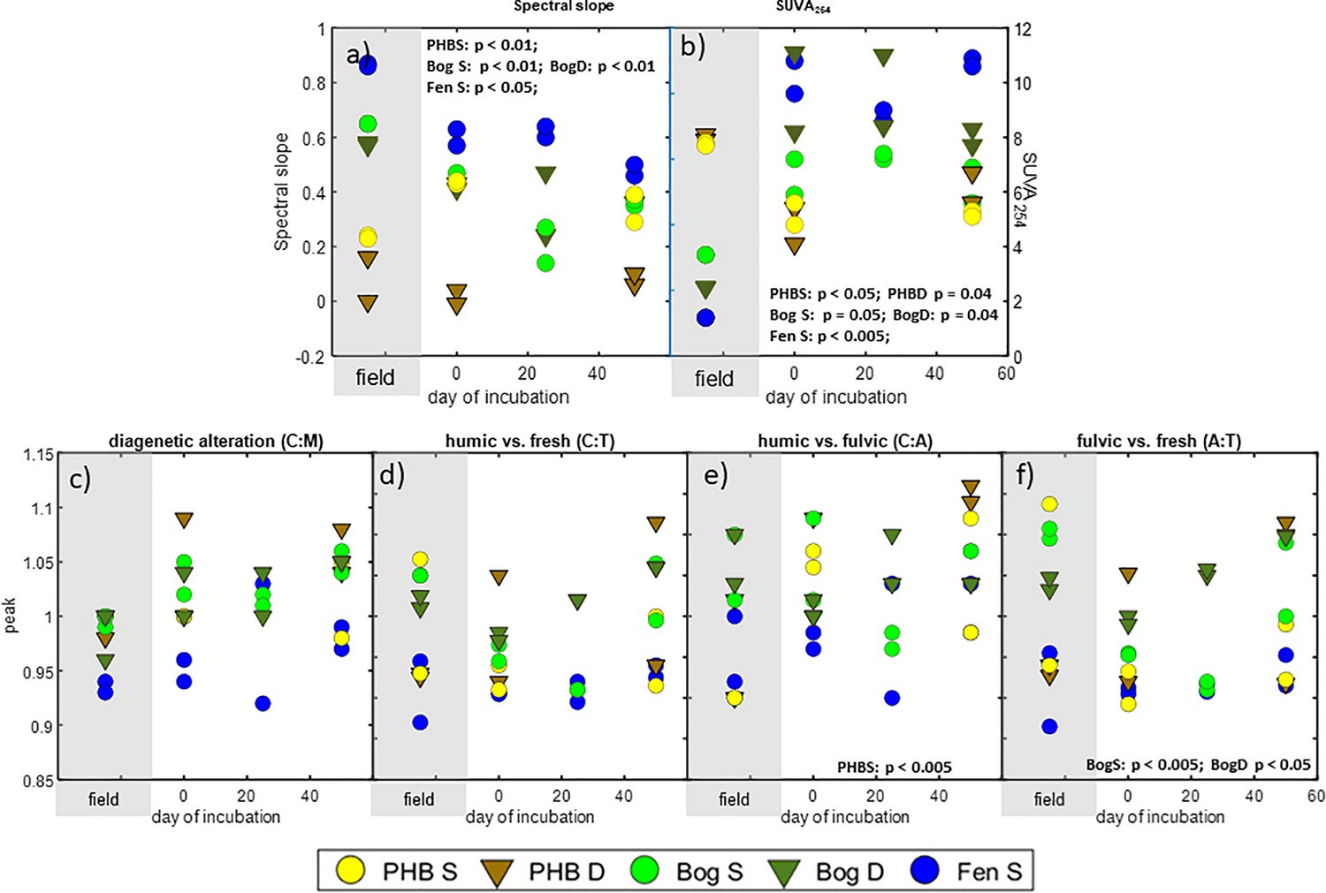
Optical measurements of bulk DOM. Individual field samples for each parameter appear in the shaded gray region on the left of each panel. Individual incubation samples are plotted against time in the white region of each plot. Both peat depths for each habitat (except fen which was not sample) are included, S indicates the 8–18 cm deep peat and D indicates the 28–38 cm deep peat. Text in each panel presents the significant results from statistical comparison of field vs. day 0 samples. Panel a) Spectral slope ratio of UV/vis results. Panel b) Specific UV absorbance at 254 (SUVA_254_; Tfaily et al. 2013) from UV-Vis analysis. Panel c) through f) provides ratios of EEMS peaks based on canonical peak areas (Coble 1996). Panel c) indicates the peak C:peak M ratio which provides a measure of diagenetic alteration. Panel d) indicates the peak C:peak T ratio which provides a measure of humic-like vs. fresh organic matter. Panel e) indicates the peak C:peak A ratio which provides a measure of humic-like versus fluvic like OM. Panel f) indicates the peak A:peak T ratio which indicates fulvic-like vs. fresh organic matter.

We have shown previously that CO_2_ and CH_4_ production in peatlands is driven primarily by substrate availability in the dissolved organic matter (DOM) pool [20,67,69,70]. Therefore, we applied a suite of complementary geochemical analyses to characterize the DOM in both the incubations and the field. The spectroscopic analyses, including ultra-violet-visible (UV-vis) and fluorescence, provide information about bulk DOM characteristics particularly with regard to molecular weight (spectral slope) and aromaticity (SUVA_254_), while the Fourier transform ion cyclotron resonance mass spectrometry (FTICR-MS) measurements provide detailed identification of individual formula comprising the DOM. Spectral slope, which is the ratio of the first derivatives of the log transformed UV-vis spectra from 275–295 nm over 350–400 nm, is inversely correlated with average molecular weight and is a commonly used metric because of the speed and wide accessibility of spectroscopy measurements. In all habitats (except the deep collapsed palsa) the spectral slope (inferred molecular weight) was significantly different in the field relative to the incubations (see Fig 3A for p-values), however, the direction of the differences varied among the different habitat types. For example, in the bog and fen, the spectral slope in the incubations was significantly lower than in the field, suggesting a lower average molecular weight (inverse relationship) in the field and that the molecular weight increased over time in the incubations. In the collapsed palsa however, the spectral slope in the incubations was significantly higher than the field, suggesting that the molecular weight decreased with decomposition of the collapsed palsa material. This may reflect differences in the starting substrate material available for decomposition from each of the habitats.

SUVA_254,_ which is the Specific UV Absorbance at 254 nm divided by the dissolved organic C concentration (*sensu*^34^), is correlated with bulk aromaticity. SUVA_254_ was higher at the start of the shallow bog and fen incubations relative to the field (Fig 3B). By contrast, aromaticity in the collapsed palsa, as indicated by SUVA_254_, was lower at the start of the incubations compared to the field. Based on SUVA_254_ and spectral slope comparisons we cannot distinguish whether low molecular weight non-aromatic compounds were produced or alternatively whether the changes during peat storage are the result of consumption of high molecular weight aromatic compounds. Similarly, the bog and fen results are consistent with either production of high molecular weight aromatics or consumption of low molecular weight non-aromatics during storage. Additional information, from the fluorescence results may help us to identify the most likely scenario to explain the SUVA_254_ and spectral slope results.

Excitation emission matrix spectroscopy (EEMS) measures the fluorescent optical properties of the bulk DOM; local excitation-emission maxima within the 3D spectra can be used to identify different fluorescent groups [40]. Integrating intensities over these defined regions and comparing peak ratios can provide information about the degree of diagenetic alteration (peak C/peak M), and the relative contributions of humic and fresh material (peak C/peak T), humic and fulvic-like material (peak C/peak A), and fulvic and fresh material (peak A/peak T) (Fig 3C–3F). There were few differences in the fluorescence ratios between the field and early incubation samples for any site; a notable exception is that there were relatively more fulvic acids in the shallow collapsed palsa field samples relative to the incubations (p < 0.005; Fig 3E). There were clear differences in diagenetic alteration among the three habitats, with the fen showing the lowest degree of diagenetic alteration (Fig 3C) and the largest contribution from fresh organic matter inputs (Fig 3D and 3F) while the opposite was generally true of the deeper samples from the bog and collapsed palsa.

Over the time of the incubations, C/M increased for all habitats consistent with our expectation that the material became increasingly altered through decomposition (Fig 3C). Similarly, the ratio of humic:fresh material as inferred from the ratio of peakC/peakT increased over time except in the shallow bog incubations (Fig 3D). This suggests that few humic substances are produced during decomposition in the shallow bog. The humic:fulvic ratio at the start of the incubations (day 0) was significantly different from the field only in the shallow collapsed palsa (Fig 3E). The fulvic fraction comprises lower molecular weight, higher O/C compounds, while the humic fraction comprises higher molecular weight (relatively) reduced compounds. From the ratio alone it is not clear which one has changed relative to the field. However, when considered together with the increasing spectral slope and declining SUVA_254_ results during the collapsed palsa incubations (Fig 3A and 3B), cumulatively these data suggest that the changes in the incubations relative to the field result from a decline in low molecular weight aromatic (i.e. fulvic) compounds. The decrease in overall aromatics (via SUVA_254_ Fig 3B) suggests that both humics and fulvics likely decreased from the field to the incubations, but the increasing C:A ratio (Fig 3E) suggests that the fulvics declined faster than the humics.

**Fig 4:**
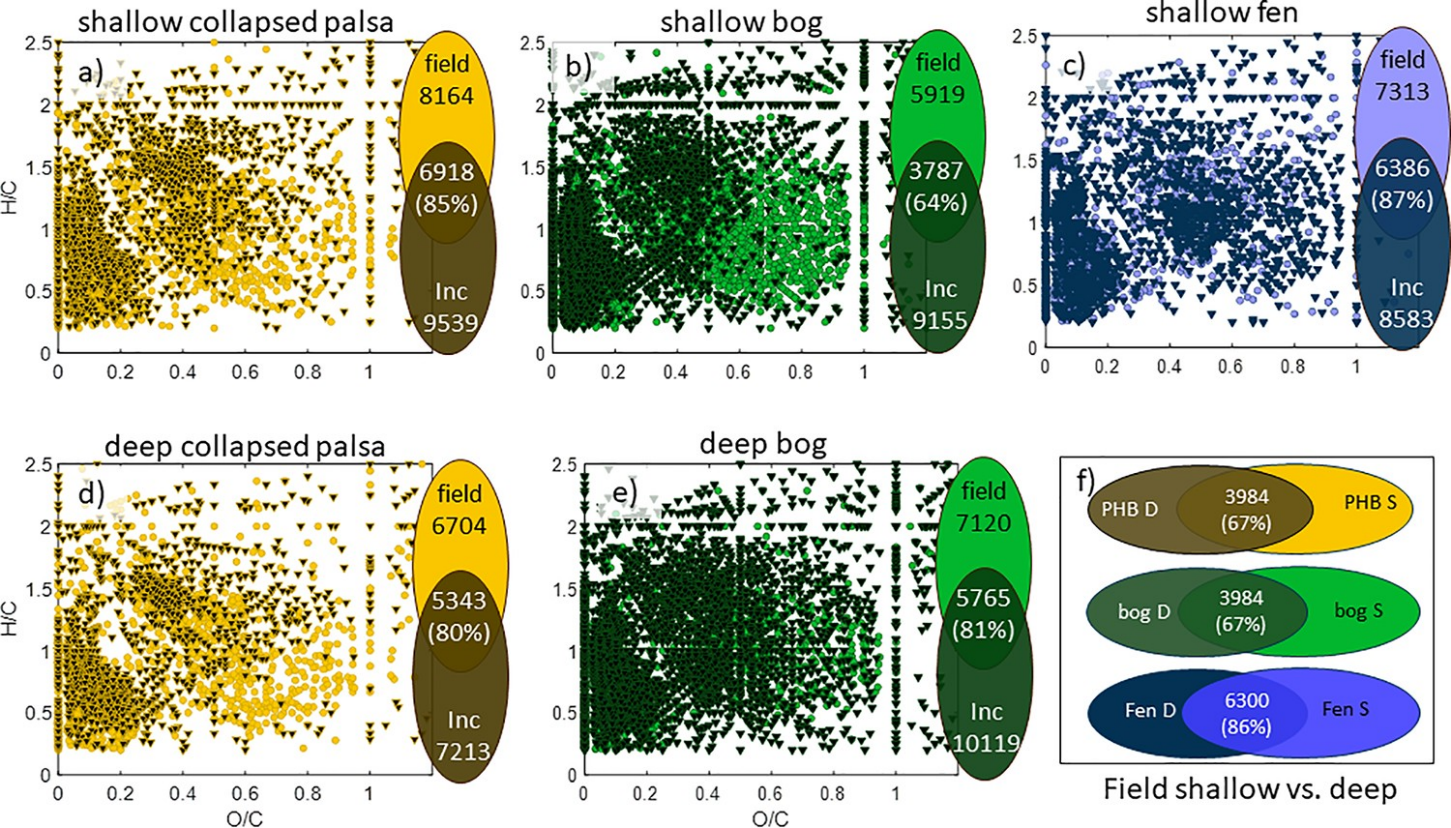
van Krevelen diagrams (H/C vs O/C ratios) of FTICRMS identified compounds for each site/depth: shallow collapsed palsa (panel a), shallow bog (panel b), shallow fen (panel c), deep collapsed palsa (panel d), and deep bog (panel e). Circles indicate compounds that are unique to the field samples for each site/depth. Triangles indicate compounds that are unique to the incubation samples for each site/depth. Venn diagrams to the right of each graph indicate the total number of compounds in the field, in the incubations, and the number of compounds that are found in both for each site/depth. The percentage indicates the percent of compounds from the field that were also found in the incubations. Panel (f) contains venn diagrams illustrating the number of samples in the shallow and deep field samples, as well as the percent of shallow field compounds that were also observed in the deep samples for each site/depth.

At the highest resolution of DOM analysis, we used ultra-high-resolution mass spectrometry (FTICRMS) to simultaneously identify thousands of individual organic compounds present in the samples (Fig 4). Although this technique does not accurately identify compounds with molecular weight less than ca. 100 Da, we nevertheless resolved >15,000 m/z peaks across all spectra, of which we were able to assign 11,255 molecular formula. This assignment rate is fairly typical of complex peatland organic matter [34].

We plotted the H/C vs. O/C ratios of assigned compounds, i.e. van Krevelen diagram, for each/site depth combination (Fig 4). In this comparison, we only show the compounds that are unique, meaning compounds that found in either the field samples or the incubation samples, but not in both. We also calculated the percent of compounds from the field that were also found in the incubations (Fig 4). The collapsed palsa incubation samples contained >80% of the compounds found in the collapsed palsa field samples, the bog incubations contained >60% of the compounds found in the bog field, and the fen incubations contained 87% of the compounds found in the fen field. This high degree of overlap between the field and incubations was similar to (and sometimes slightly better) than the overlap between shallow and deep samples collected at the same site (Fig 4F). This suggests that the complexity of the DOM from the field sites is preserved in the incubations allowing us to probe reactions of these secondary metabolites during the incubations. In the collapsed palsa and bog, compounds in the lower right of the van Krevelen diagrams appear to have been lost relative to the field, while unique compounds produced in the incubations are shifted to the upper left. This shift towards higher H/C and lower O/C ratios is consistent with a reduction of the organic matter in the incubations relative to the field as would be expected from continued decomposition. The unique compounds in the field and incubations from the fen don’t have any obvious pattern suggesting that, not only is a large percentage of the field compounds also present in the incubations, but the overall distribution of compound classes is also similar to the field samples. Notably, the FTICRMS data identifies compounds present but does not quantify them, meaning the relative importance of the unique versus shared formulae, in terms of their proportion of the total DOM pool, cannot be assessed.

**Fig 5:**
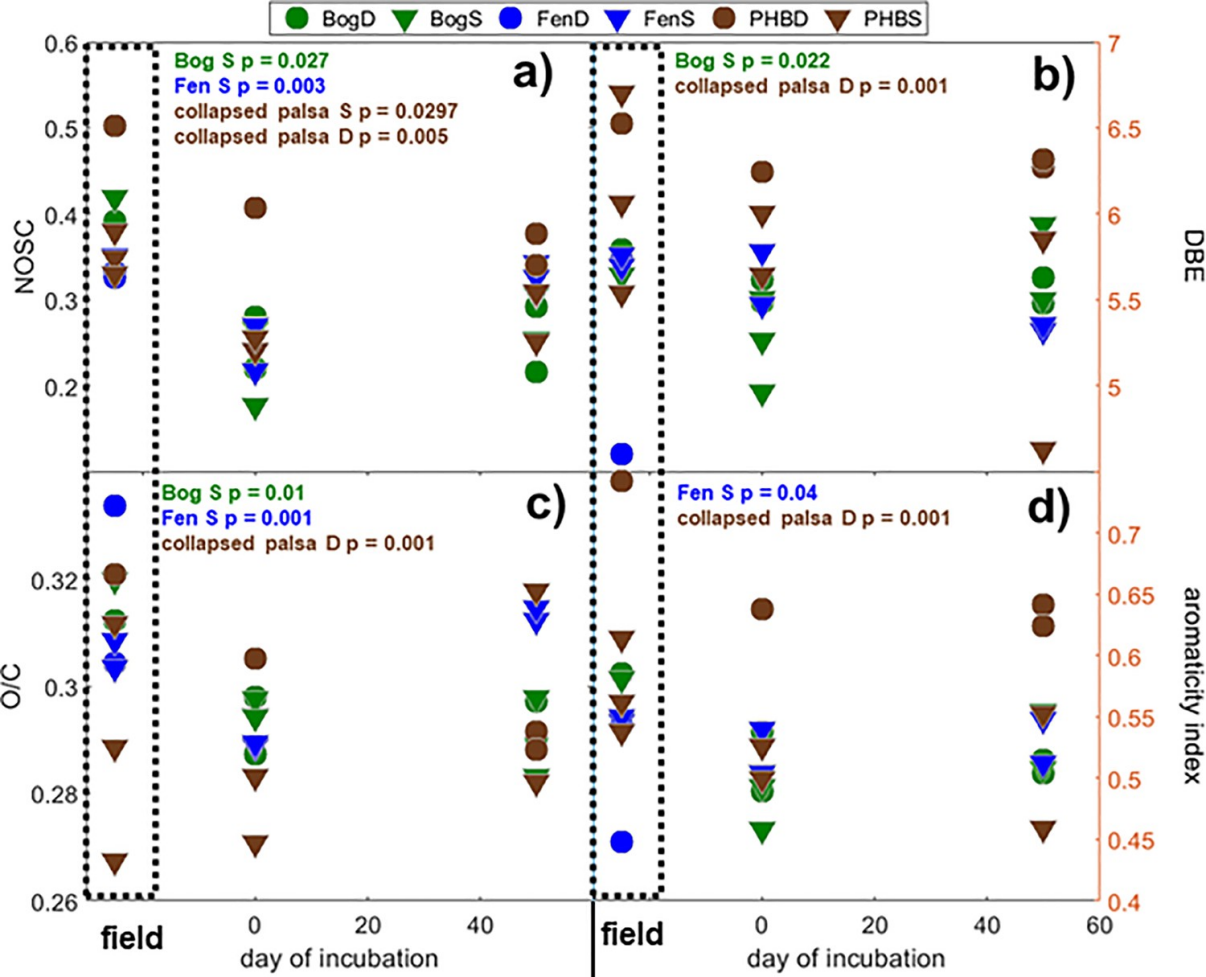
DOM characteristics identified by FTICRMS. In panels a-d: field results are outlined by dashed boxes and plotted preceding day 0 and day 50 of incubation. Text indicates results of a t-test comparing field and day 0 incubation samples. Symbols indicate average for each characteristic; inverted triangles are the 8–18 cm samples and circles are the 28–38 cm samples from each site. Panel a) denotes the nominal oxidation state of carbon (NOSC), panel b) denotes the double bond equivalence, panel c) denotes the oxygen to carbon ratio, and panel d) denotes aromaticity index.

From the FTICRMS-identified formulae, we are able to calculate a number of measures of organic matter quality. The average nominal oxidation state carbon (NOSC; calculated according to [71]) reflects the remaining potential energy yield during decomposition [72] and, therefore, provides a measure of organic matter quality [73]. In general, average NOSC were lower in the incubations than in the field for a given site (Fig 5A), this is consistent with the more extensive degradation in the DOM of the incubations resulting in lower quality organic matter and is consistent with the trend of more reduced compounds in the incubations relative to the field observed in the van Krevelen diagrams (Fig 4). The NOSC decline was significant in all incubations except the deep bog where overall decomposition rates (as evidenced by CO_2_ and CH_4_ production, Fig 1) were lower than in the other samples. Aromaticity measured by double-bond equivalents was lower in the bog and deep collapsed palsa incubations compared to the field consistent with earlier observations of hydrogenation of organic matter acting as a terminal electron sink through the reduction of double bonds in anaerobic peatlands [32].

**Fig 6:**
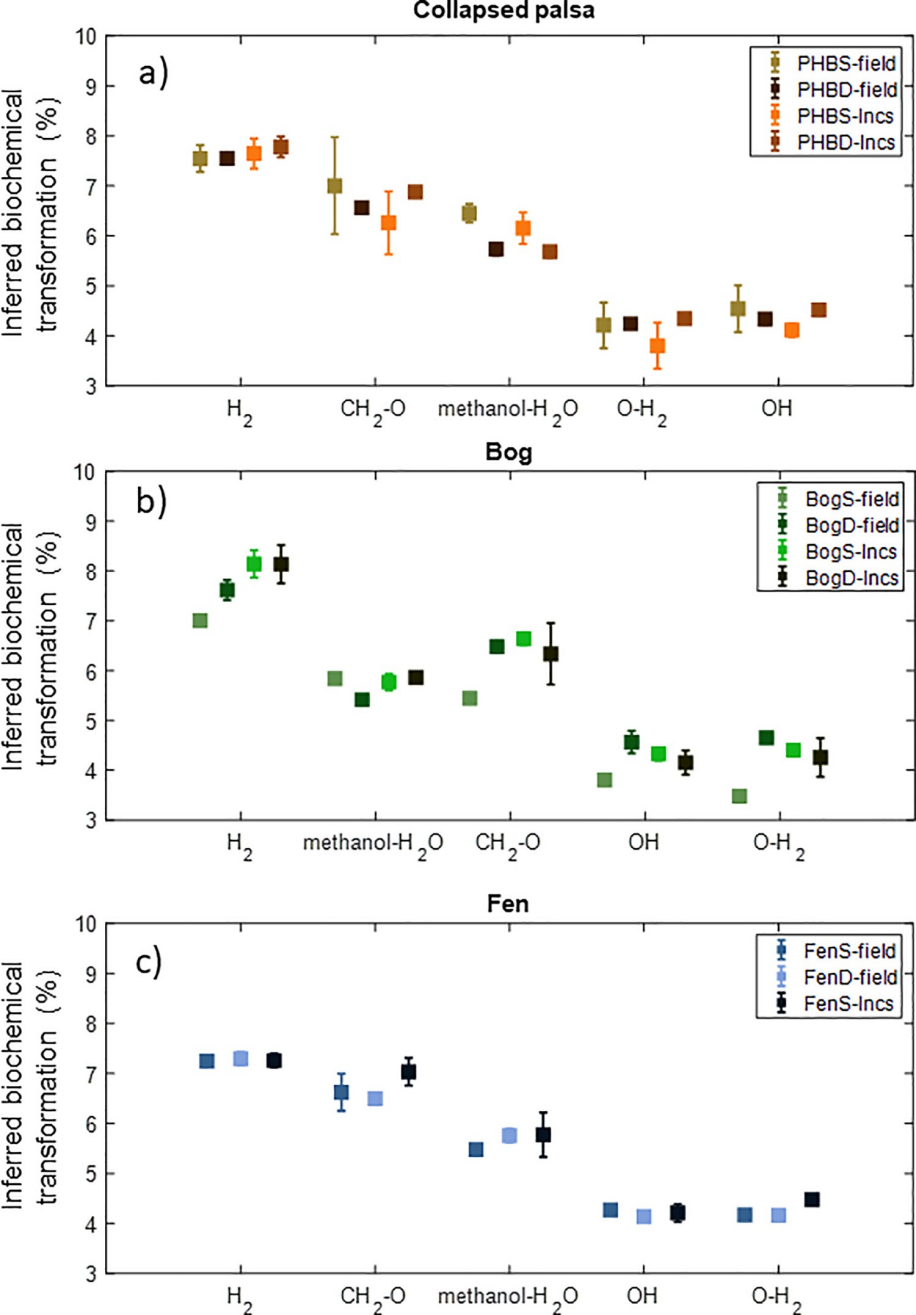
Top five transforms by percentage for each habitat: collapsed palsa (panel a), bog (panel b), and fen (panel c). Squares indicate means of replicates of the shallow field, deep field, shallow incubations and deep incubations results respectively, with whiskers denoting 1 standard deviation. Counts for all transforms identified in each sample are provided in S2 Table.

Another useful metric that can be calculated from the ultra-high resolution mass spectrometry data are the transformations or chemical changes that individual molecules undergo during microbial decomposition. Because of the ultra-high resolution of this technique it is possible to identify transformations that result in specific chemical moieties being gained or lost over time from individual compounds. To do this, we calculated the mass differences between all compounds within a sample following the mathematical procedures described elsewhere [32,74]. We then compared the resulting mass differences to an in-house curated reference database of (currently) 185 chemical moieties assembled from the literature that represent potential microbial and abiotic decomposition strategies. For example, a mass difference between two compounds of 43.98983 Da corresponds to a loss of CO_2_ and is interpreted as decarboxylation. This comparison was used to identify the dominant chemical transformations (i.e. gain or loss of a specific chemical moiety) by which compounds were being transformed in the field as well as in the incubations (Fig 6). It should be noted that each individual compound may be involved in multiple transforms, meaning it can be chemically altered in different ways, thus the sum of transform counts could be greater than the number of identified compounds in each sample.

The top five transforms accounted for ~30% of the total number of transforms and were similar in all three habitats, though their rank of importance differed slightly among all three sites. In comparing the field and incubation transforms within each habitat type, the percentages for each transform type are overall very similar, typically within 1 standard deviation overlap, and notably follow similar rank order (i.e. H_2_ > methanol-H_2_O > OH, etc) within each habitat type. Considered together with the high percent of compounds identified in the field that are also in the incubations samples, this suggests that despite differences in some of the bulk characteristics of the organic matter, the transformations, and therefore, decomposition processes for secondary metabolites (>100 Da) are surprisingly similar in the field and incubation samples. Thus, we can infer that chemical mechanisms working on the secondary metabolites occurring in the incubations largely reflect the dominant transformation mechanisms in the field.

#### 3.1.3 Solid phase organic matter changes

**Fig 7:**
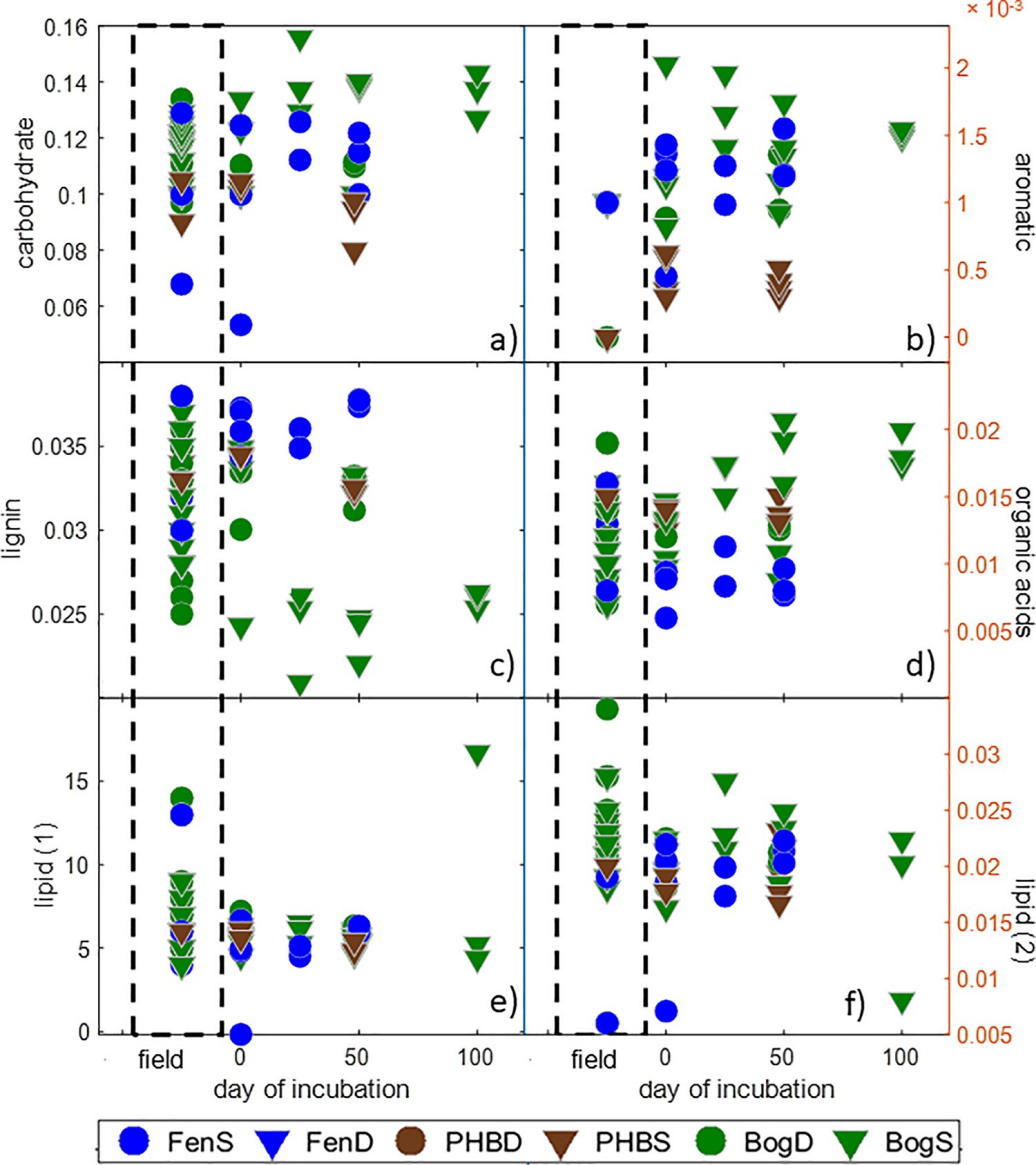
Solid-phase peat chemical functional group composition comparison for surface samples. FTIR results showing the baseline-corrected peak areas of carbohydrates (1030 cm^-1^, panel a), lignin-like aromatics (1510 cm^-1^, panel c), aromatics and deprotonated carboxylic acids (1630 cm^-1^, panel b), organic acids (1720 cm^-1^, panel d), and lipids (2850 cm^-1^, 2920 cm^-1^, panel e and f, respectively) as percentages of total spectral area. Day 0 of the incubations was not different from the field for any parameter (Anova, p > 0.05). The different depth peat are indicated by symbol shape, shallow peat are circles, and inverted triangles are for the deep peat incubations.

Although the dissolved phase of OM has been shown to be the primary driver of the microbially-mediated reactions that give rise to CO_2_ and CH_4_ production dynamics [20,67,69,70,75], the solid phase represents a potentially large C pool that may also play a role in C emissions [76,77]. Based on Fourier Transform Infrared Spectroscopy (FTIR) analysis, the relative contributions of lipids, carbohydrates, aromatic compounds, lignins and organic acids in the solid peat material were similar in the field and incubations (Fig 7). The high similarity between the field and incubation solid phase measures is not surprising given that the composition of the solid phase could be expected to change more slowly than the dissolved phase *ex situ*. Inter-habitat differences in bulk solid peat chemistry appeared to be dominated by differences in the plant communities. For example the bog had lower lignin content than in the fen as is expected in *Sphagnum-*dominated sites since *Sphagnum* mosses do not contain true lignin.

### 3.2 Microbial community results

#### 3.2.1 Microbial cell abundances

**Fig 8:**
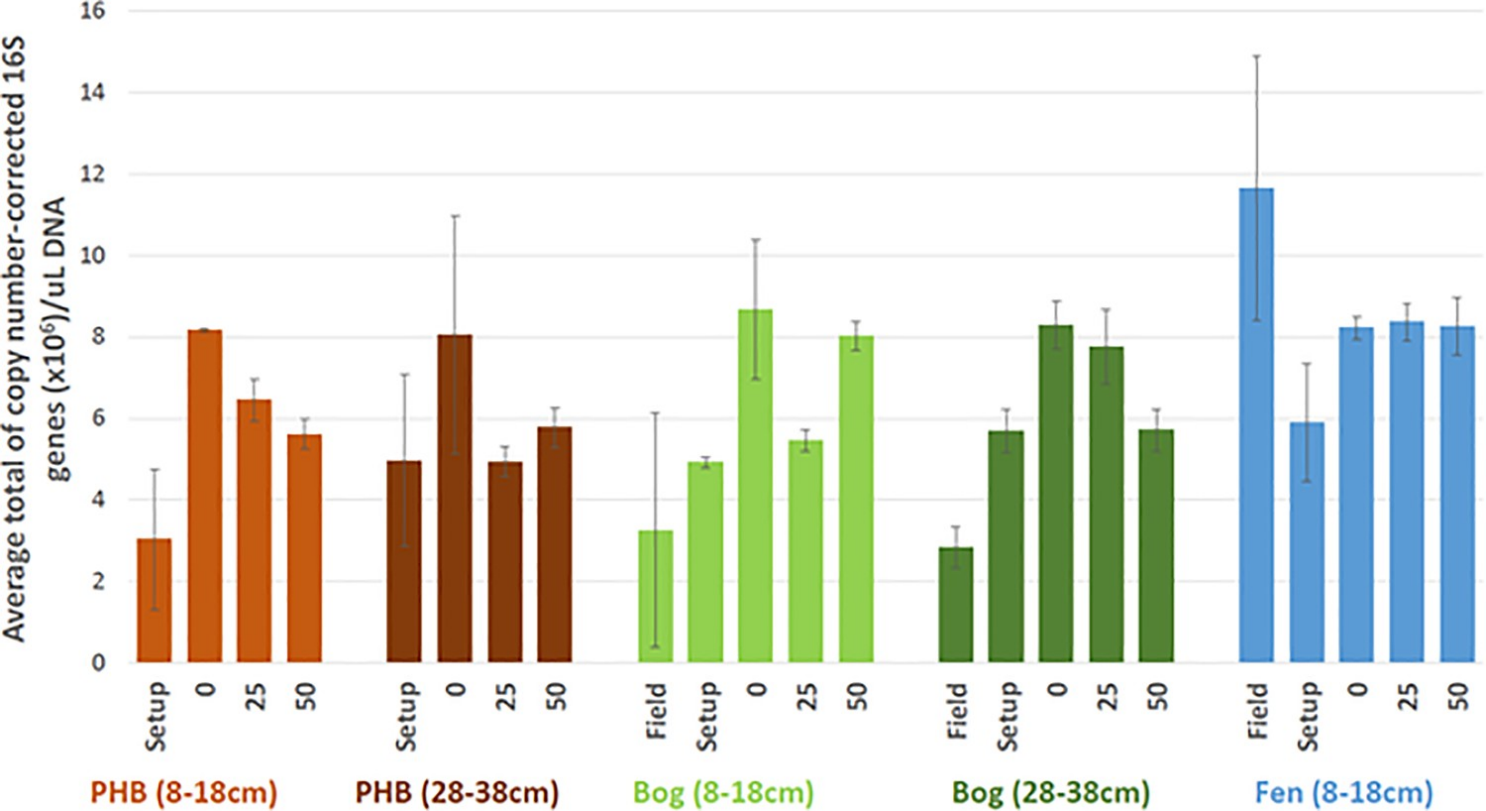
The cell concentrations (by copy-corrected 16S rRNA counts; and per ul of extract) in field and incubation samples, by quantitative polymerase chain reaction. Two replicates were averaged at each time point and error bars represent standard deviation. ‘Setup’ denotes post-storage, at the establishment of pre-incubations. 0, 25 and 50 refer to the elapsed incubation day. Matched field samples of identical depth range were missing for PHB.

To infer the abundance of microbial cells, the 16S rRNA gene abundances were measured, and corrected using the community composition profiles (below) for known variation in 16S rRNA gene copy number among microbial lineages (see Methods; due to sample loss, field-v-incubation comparisons were possible for shallow and deep bog and shallow fen samples only, for all molecular analyses, and incubation time-course analyses were possible for all habitats except fen-deep). In general, the inferred cell concentration increased during the storage period, increased further during the pre-incubation, and then decreased through the incubation time (Fig 8); this trend was also present in uncorrected 16S rRNA concentrations. The increase in cell numbers during storage could have been due to the creation of more heterogeneous, partially oxic conditions allowing the proliferation of aerobes, and the pre-incubation increase in cells could have been due to the temperature increase (2°C storage to 18°C incubation) speeding metabolism and cell proliferation. The subsequent decrease in total cell number over incubation time was accompanied by decreases in diversity, likely due to being in a closed system without replenishment of nutrients or food sources, and the associated decrease in substrate energetic “quality” reflected in the DOM characterizations (decreasing NOSC, Fig 5). However, few of these temporal changes were statistically significant (S3 Table), and overall field-vs-incubation shifts (all incubation days combined) were only significantly different (p < 0.05) for the deep bog (and were significantly *higher* in the incubations in that case; this might have been due to inhibitory compounds release *ex situ* from live Sphagnum, though we would expect that effect to be stronger in the shallow bog). Thus, though there were visible trends in the inferred cell numbers, they were not generally significantly different in the incubations compared to the field.

#### 3.2.2 Alpha diversity

Community profiles from 16S rRNA gene amplicon sequencing recovered 1538 distinct microbial lineages across all samples. The communities appeared well-sampled at the sequencing depth and clustering applied, based on leveling rarefaction curves for most samples. Post-processing reads per sample averaged roughly 40,000 (min ~7,000, max ~145,000). While community functional profiles predicted from 16S rRNA composition via PICRUSt are imprecise (particularly for habitats with less genomic representation in utilized reference databases), several studies have demonstrated ~80% agreement with those inferred from metagenomes across diverse soil environments [78,79]. In addition, these 16S rRNA-based functional inferences are more robust for early-evolving microbial traits such as carbohydrate and energy metabolism rather than functions involved in, for example, host-phage interactions, and we focus here on the former. We calculated alpha diversity metrics (richness, Shannon diversity index, and Pielou’s J evenness metric) to compare field versus incubation community characteristics (Fig 10). As with the cell numbers, alpha diversity metrics increased over the storage period in the bog and shallow fen where it could be assessed (S4 Table). These increases are consistent with the hypothesized creation of more heterogeneous redox conditions during refrigerated, bagged storage allowing more rare lineages to rise to the level of detection. During the pre-incubation period, in contrast to the increased cell numbers, alpha diversity generally decreased. This implies that a subset of lineages reproduced more rapidly as the peat warmed, and anaerobic conditions were established, during preincubation such that previously observed lineages were pushed below observation even as overall cell numbers increased.

During the incubation period itself, in all habitats and depths, richness and diversity decreased over time (S4 Table) as it had during pre-incubation. The magnitude of the temporal trends was inversely related to thaw stage–sharpest in collapsed palsa, and then bog, then fen. This progression could suggest that, in the closed incubation system, the microbial communities may have used up labile organic matter present in the collapsed palsa earliest, leading to a quicker community simplification, followed by the bog, and then the fen, where the most labile compounds were present in the initial field samples (and where only richness decreased with time, while diversity and evenness did not). However, the constant production rates over time are not consistent with labile substrate limitation (Fig 1) nor are the observed changes in diagenetic alteration or humic:fresh ratios in the DOM (Fig 3C and 3D). An alternative explanation is that microbes in the collapsed palsa experience the most aerobic conditions in the field, and therefore may not be as well adapted as bog and fen microbes to survive in the anoxic incubation conditions. Overall, the microbial communities derived from samples from the deeper depths shifted less in alpha diversity metrics over time as compared to the shallower samples. We hypothesize that the deeper communities experience more anaerobic conditions in the field, and were therefore altered less by the anaerobic incubation conditions.

**Fig 9:**
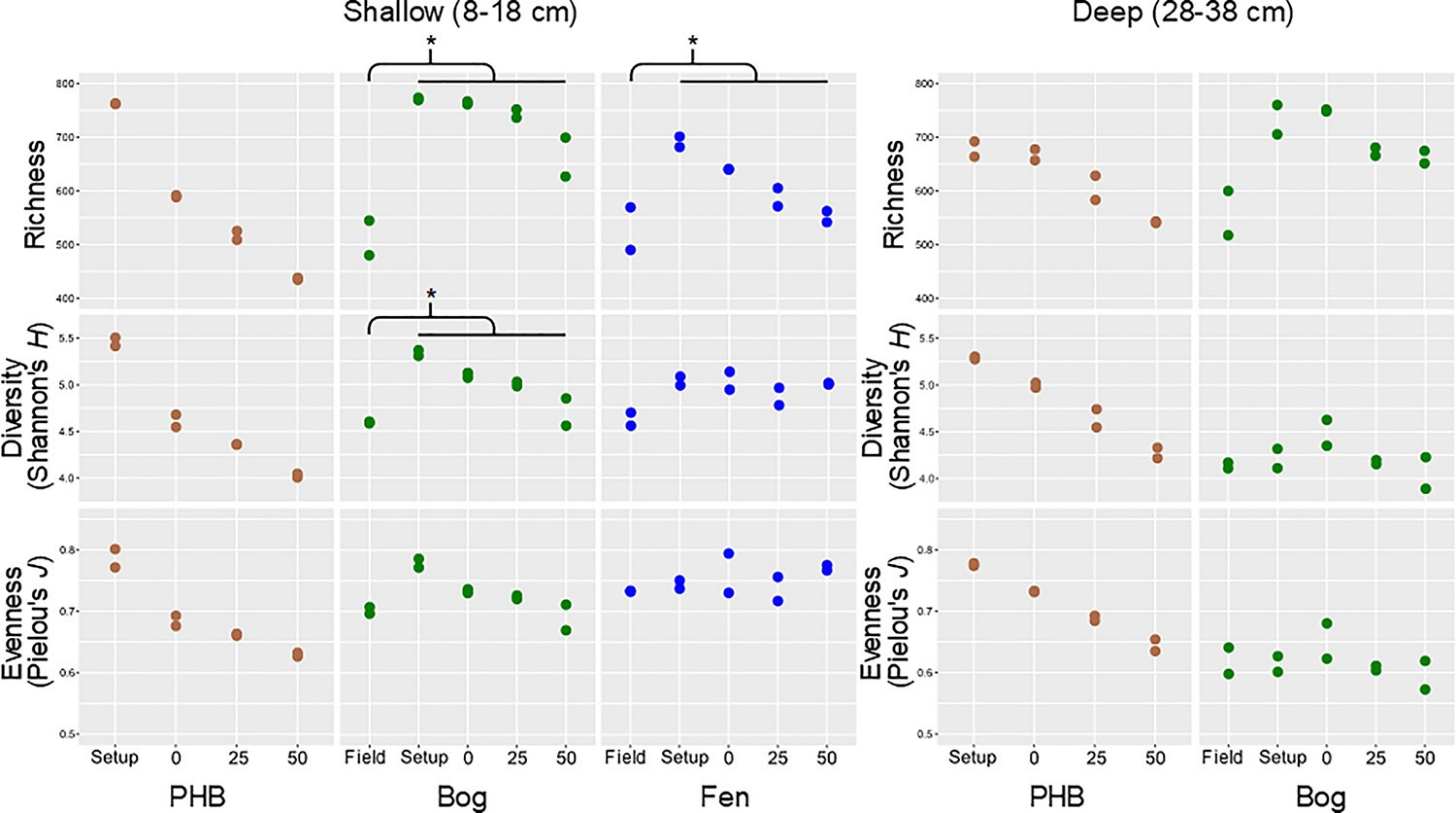
Alpha diversity metrics of microbial communities at field collection, at incubation setup (post-storage), and over incubation time within each habitat/depth. An asterisk indicates a significant difference (p-value < 0.05) between the field sample and the average of the incubation (including the incubation setup) samples. The significance of these differences is reported in S4 Table.

In spite of the temporal dynamics from field through storage, preincubation, and the course of incubation, the overall field-vs-incubation differences (all incubation days combined) generally showed *higher* diversity metrics for the incubation samples (Fig 9). This increase in diversity, even as cell numbers generally decreased, was unexpected, and stands in contrast to the observed simplification of aquatic incubations [80,81]. We hypothesize that the much higher microscale habitat heterogeneity of soils relative to most aquatic systems buffers them from the onset of ‘bottle effects’. Indeed, substrate diversity remained high, with numerous new compounds unique to the incubations appearing even as a subset of field compounds disappeared (note the number of compounds in incubations compared to the field in Fig 4). A second potential cause of the high diversity in the incubations is suggested by the fact that the only incubations that were *significantly* different than the field (S4 Table) were the shallow bog for richness and diversity. As with the cell numbers in the deep bog, we hypothesize that this increase was due to separation from living *Sphagnum*, which can release compounds that inhibit microbial activity [82] and lower the environmental pH [83], allowing a higher diversity of microbial lineages to proliferate. Although these samples (at 8–18 cm and 28–38 cm) were appreciably below the depth of the surface-dwelling *Sphagnum* spp. [84], the removal of hydrologic connection to living *Sphagnum* in the incubations may ameliorate the moss-associated inhibition of the microbes.

Higher diversity has been hypothesized to confer more stability, and offers more opportunity for functional redundancy [85], and indeed the bog incubations, which were the most diverse, also showed the most geochemical similarity to the field of the three habitats. The response of microbial communities to disturbance depends on the type of disturbance [13], interactions with the environment, and history of previous disturbance [85]. Thus, redox disturbances in the bog field site, resulting from water table fluctuations, may have allowed the bog microbial community to develop functional redundancy, enabling more complete recovery from aeration during storage.

#### 3.2.3 Phylum-level community differences

**Fig 10:**
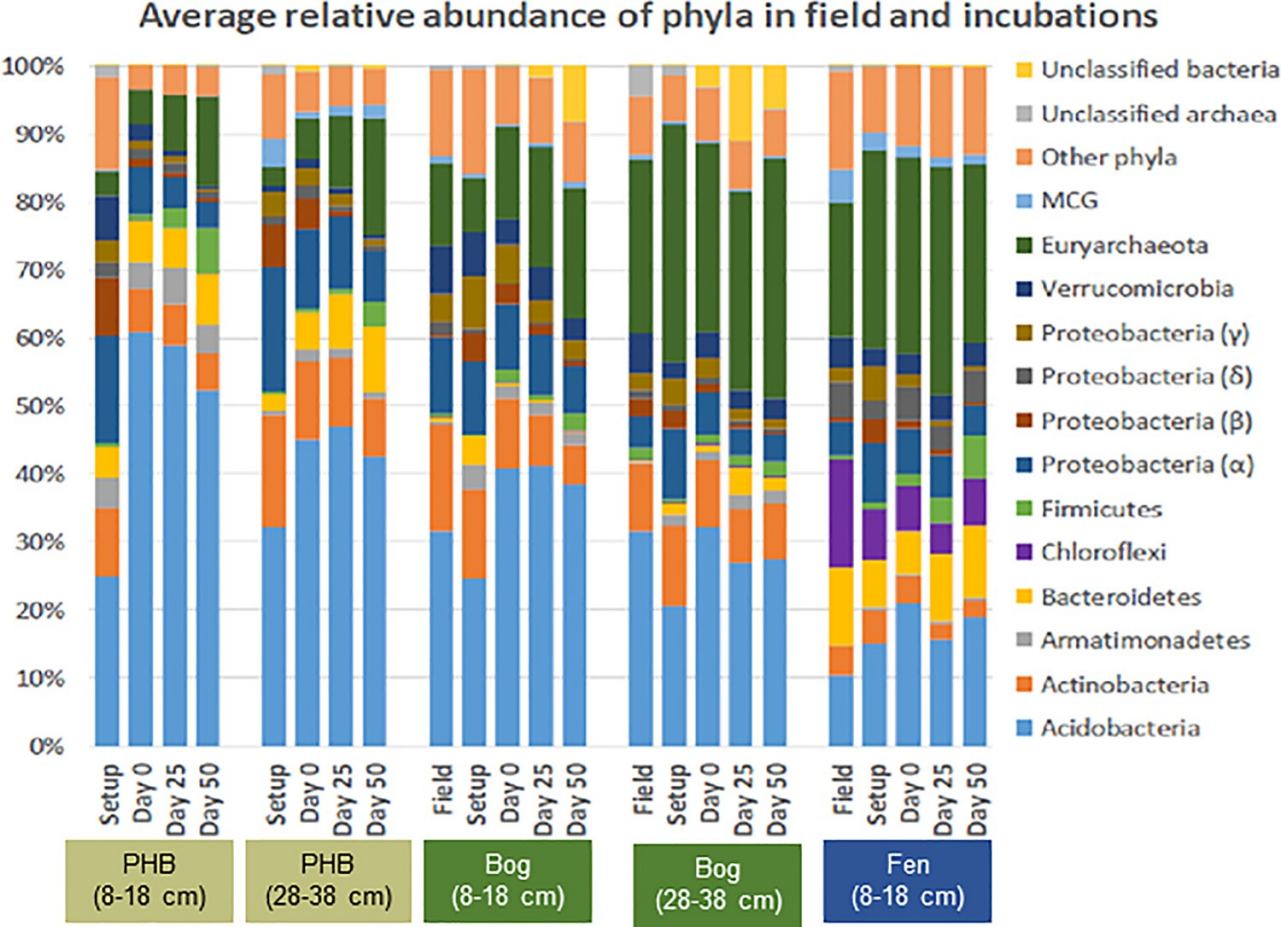
Relative abundances of microbial phyla in field and incubation samples. Relative abundances were averaged between duplicates at each time point. “Other phyla” were microbial phyla below 1% relative abundance. “Unclassified” bacteria and archaea were OTUs that did not have a BLAST-assigned taxonomy at the phylum level.

To characterize high-level community differences among samples, dominant phyla (classes for *Proteobacteria*) (as duplicate averages for each time point) were examined (Fig 10). Eleven phyla were present at > 1% relative abundance in at least one sample, with *Acidobacteria* consistently a dominant phylum in all sites, depths, and time points, though decreasing in relative abundance across the thaw gradient with depth. These trends were consistent with previous work which showed that, in the field, *Acidobacteria* were ubiquitous at this site and were more abundant in palsa and bog than fen [45,86]. A large portion of known *Acidobacteria* are aerobes [87], suggesting the potential for novel anaerobic Acidobacterial lineages at this site. In all habitats and depths, the relative abundance of *Acidobacteria* increased during the pre-incubation period, with the largest difference in the collapsed palsa communities, which also were likely to experience oxygen more frequently *in situ* and thus potentially have more aerobic lineages that would be lost during the pre-incubation phase. *Bacteroidetes* was also abundant, highest in the fen and least in the bog. Members of this phylum have been demonstrated or predicted to ferment plant-derived hemicellulosic sugar monomers in anaerobic rumen systems [88,89], and these substrates were likely similar to the plant material at the Mire. *Actinobacteria* were similarly abundant in collapsed palsa and bog but decreased in relative abundance by about a half in the fen.

#### 3.2.4 Beta diversity

**Fig 11:**
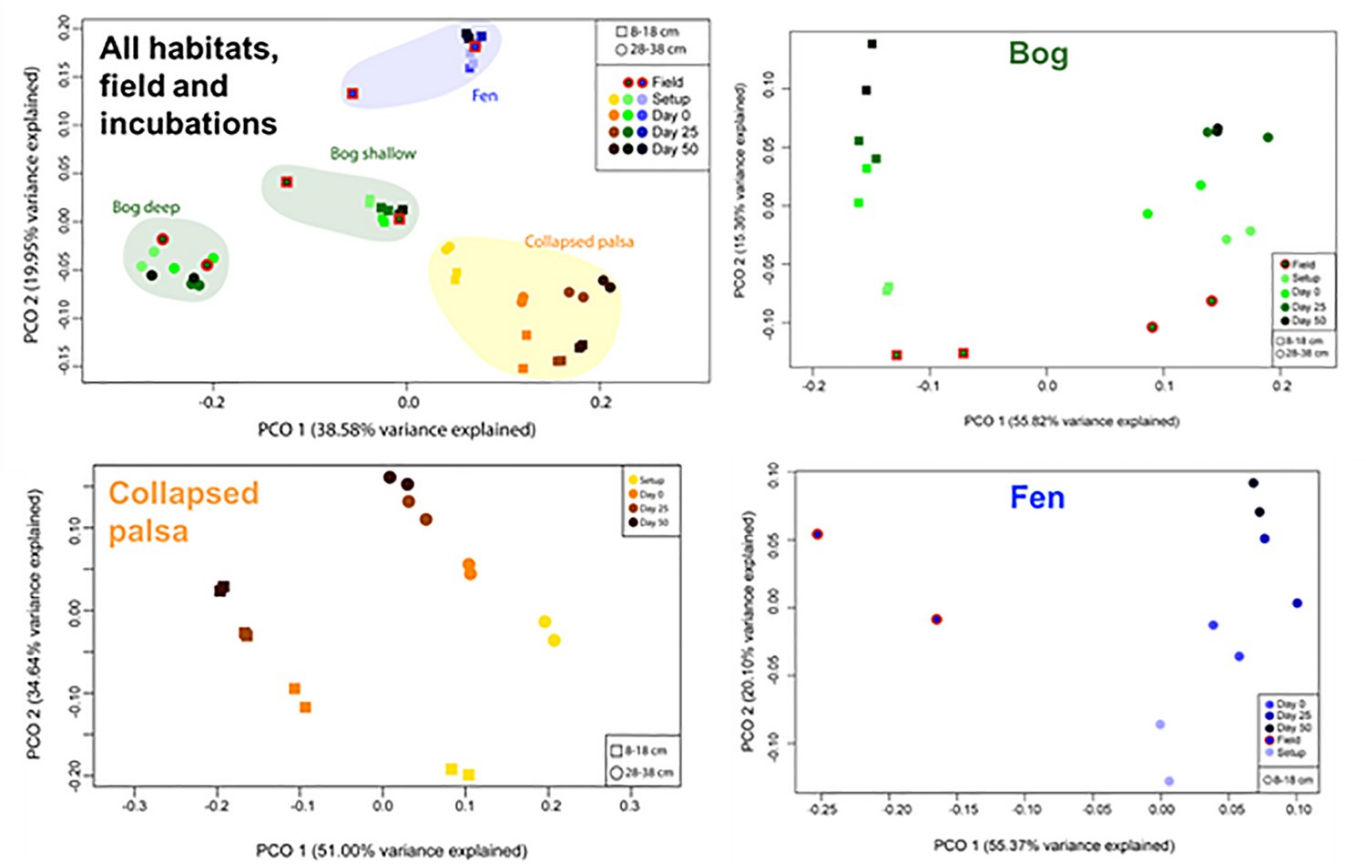
**Principal coordinate analysis of microbial communities by weighted Unifrac distances, with samples identified by habitat (collapsed palsa as yellow/orange/brown, bog as green, fen as blue), depth (shallow as square, deep as circle), and field (red outline) versus increasing time since collection/incubation (darker shades of each habitat color).** Habitat-specific principal coordinates analysis of microbial communities by weighted Unifrac distances top: collapsed palsa middle: bog; bottom: fen).

To compare the microbial communities at the highest-resolution out level, weighted Unifrac distances (which account for both relative abundance and phylogenetic distance) were calculated between all communities in this study and used to represent community composition by principal coordinates analysis (PCoA; Fig 11). Samples were separated primarily and significantly by habitat (ANOSIM: R = 0.7662; p = 0.001), and by depth for the bog. The collapsed palsa communities also shifted directionally along PCo1 with time since collection. PCo2 separated samples based on ombrotrophic (collapsed palsa and bog) vs. minerotrophic (fen) status. The overall strong separation of samples by habitat and along trophic status, even throughout the anaerobic incubations, is consistent with previous field-based observations of robustly distinct communities along the thaw gradient [86].

**Fig 12:**
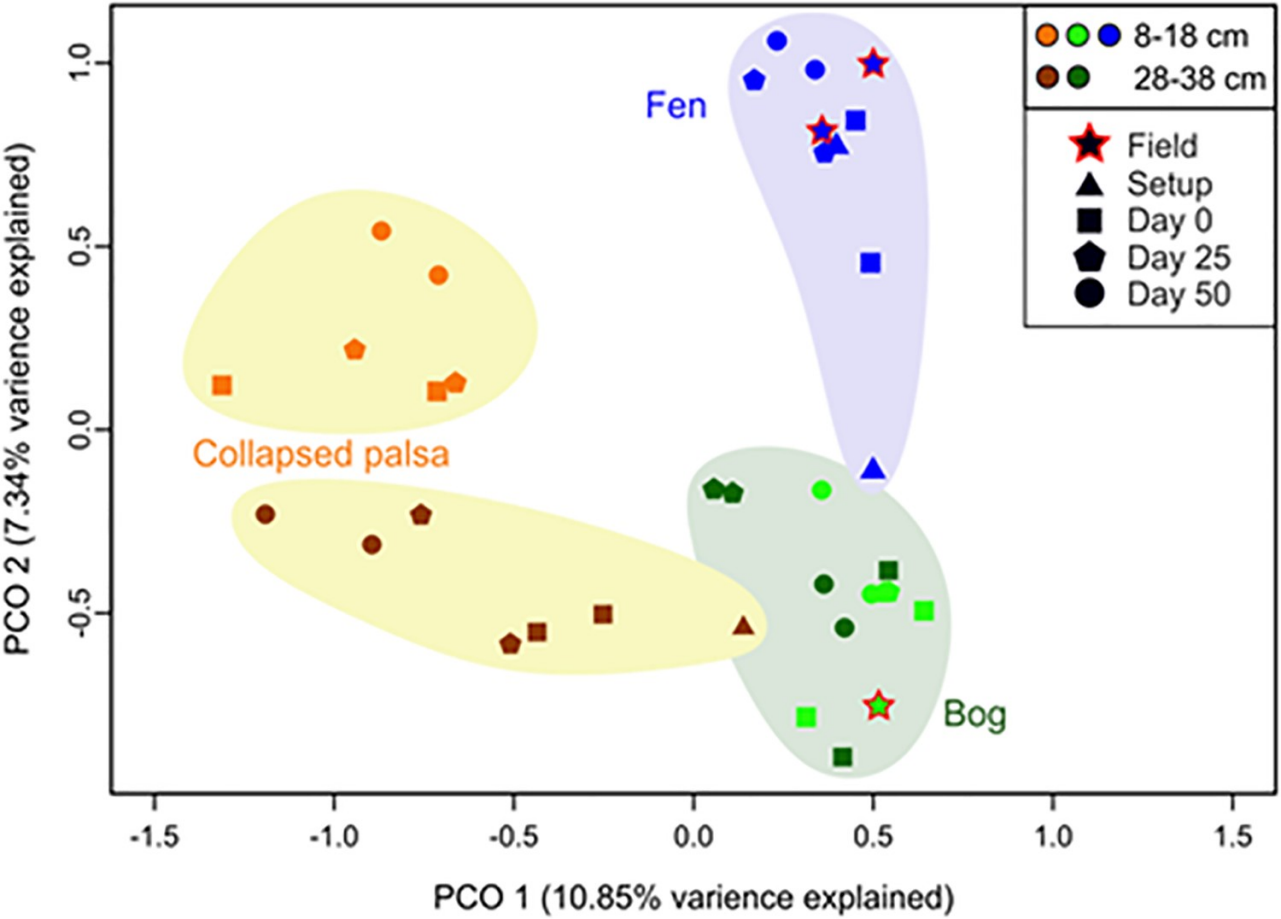
Ordination and metabolic pathways for PICRUSt-predicted microbial community functions. Principal coordinate analysis of predicted microbial community functions, with samples identified by habitat (collapsed palsa as yellow/orange/brown, bog as green, fen as blue), depth (shallow as square, deep as circle), and field (red outline) versus increasing time since collection/incubation (darker shades of each habitat color).

We also examined each habitat separately to remove the overriding impact of the habitat differences (Fig 12). After habitat was removed, depth was the clear secondary driver of community differences, and in each case, communities changed with time since collection, in a generally linear fashion. This could be due to the decreases in overall diversity (Fig 9), such that each habitat retains its distinct composition over time but with a decreasing number of lineages present.

**Fig 13:**
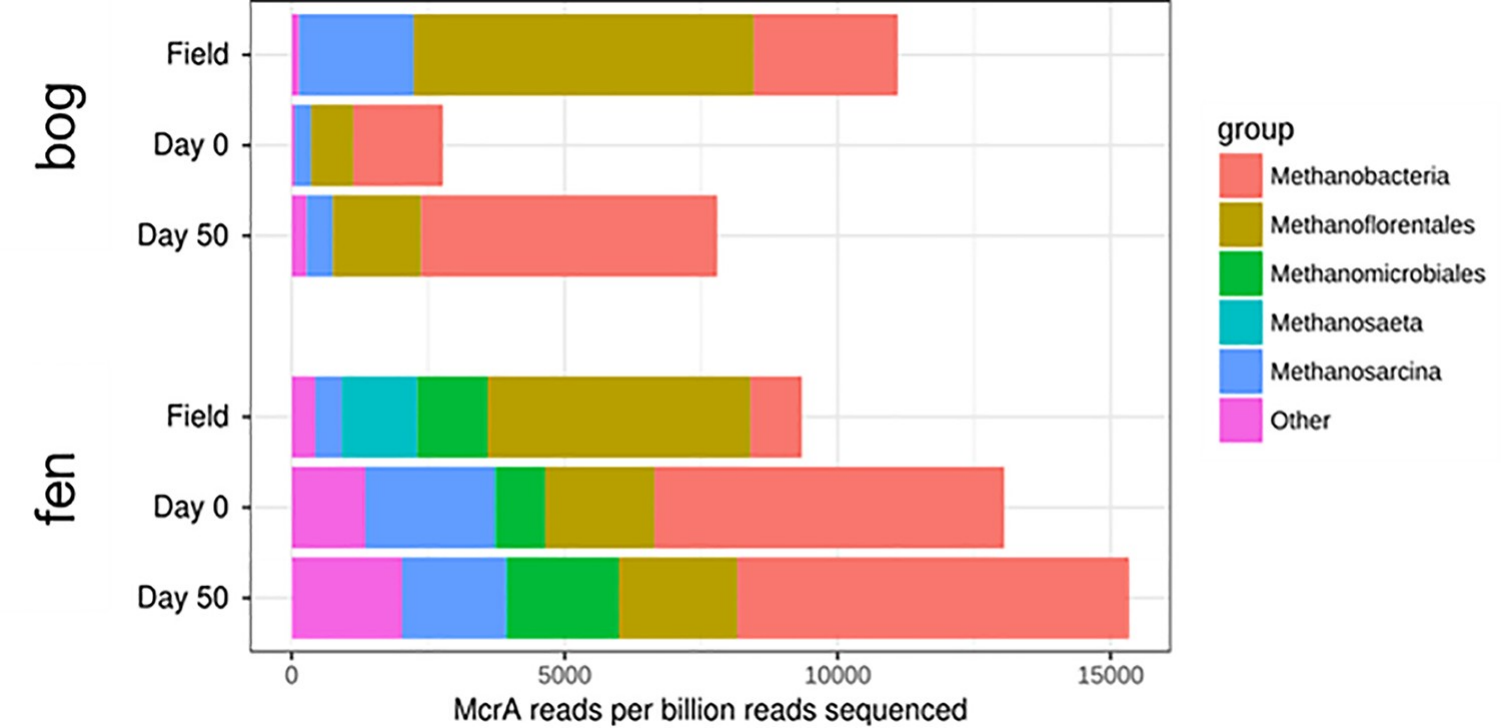
Methanogen abundance and diversity in bog and fen field and incubations, via the methanogen marker gene *mcrA*.

Similar to the results from OTU ordination, principal coordinate analysis of the rarefied functional profiles predicted from the 16S rRNA sequences (see Methods) revealed that samples were separated primarily by habitat (except for one setup fen sample and one deep setup collapsed palsa sample), and by depth for the collapsed palsa (Fig 13). Additionally, the deep and shallow collapsed palsa communities shifted along PCo1 and PCo2, respectively, with time since collection. However, the bog’s depth partitioning at the OTU level was not observed at the predicted functional analysis level suggesting that the different taxa observed at these two distinct depth categories are likely contributing to the same general community function (i.e. are functionally redundant). Moreover, the ombrotrophic vs. minerotrophic separation along PCo2 that was observed at the OTU level was to a lesser extent maintained at the predicted functional level. This implies that differences in the peat matrix of each habitat were a greater driver of variation in resident microbiota and their collective functions than short-term treatment, at the scale of these storage and incubation conditions.

To look at the functional profiles of these microbiota at higher resolution, we examined the individual KEGG metabolic functional modules for each rarefied sample with at least 400 predicted KEGG functions (see Methods). A heatmap of the predicted microbial community functions clustered by their metabolic pathway has been provided (S1 Fig). Relative abundances were averaged between duplicates at each time point before summing all the relative abundances of the KEGG functions per pathway for the same time point. Abbreviations: S; Shallow, D; Deep. Both analyses were conducted after rarefying each sample down to 500 KEGG functions, excluding those with very low number of predicted functions (<400 KEGG functions). The results of this analysis (Fig 13) suggested that the majority of these functional modules were maintained at the same abundance level across the timepoints and between the field and incubation samples for the same habitat and depth category.

#### 3.2.5 Pairwise community comparisons

The statistical significance of community compositional differences was assessed within each habitat and depth for all comparisons (field if available, setup, Day 0, Day 25, Day 50, as well as the collective incubation Days 0, 25, 50) via PERMANOVA of the weighted Unifrac distances (Fig 13). The 4-month partially aerobic storage period did not significantly change the communities present in the bog (shallow and deep) or fen (shallow) (p > 0.05), but the ensuing anaerobic incubations did (p < 0.01 comparing the field community to the collective (Fig 13: Days 0, 25, and 50 communities, and p < 0.05 for the setup relative to the collective incubations). In the collapsed palsa where paired field samples were unavailable, the incubation communities were significantly different from those post-storage (p < 0.01 for both depths, comparing setup to the collective Days 0, 25, and 50 communities). While the pairwise comparisons of field or setup to individual incubation time points generally were not significant, comparisons to the collective incubation data generally were, likely due to the higher number of samples improving statistical resolution (only two replicates were available at each time). One exception to this was in the collapsed palsa samples, for which the setup samples were significantly different than Day 0; this could be due to more generally oxic field conditions in the collapsed palsa, resulting in larger change when transitioned to fully anoxic incubations.

Previous work assessing storage impacts on microbial communities from soil, human skin, and human guts, after much shorter sample storage than ours (3 or 14 days) and a range of temperatures (-80°C to 20°C), found no significant impact [90]. However, in our work, the storage time was much longer and the temperature prior to incubating the peat material was within the range experienced in the field site. Thus, over a 4-month partially oxic storage we had hypothesized that changes in the microbial community would have occurred. The lack of significant impacts on community composition in our study’s bog and fen samples from the long-term (~4 month) refrigerated storage period prior to the incubation experiment was somewhat unexpected. It is not uncommon for soil samples to be stored at ~4°C between collection and incubation, as this temperature is typically low enough to slow microbial metabolism. Other studies have examined soil storage effects on measured enzymatic activity, substrate utilization, and overall microbial biomass, rather than community compositions. Gonzalez-Quiñones et al., [90,91] examined community-level physiological profiles using MicroResp from four distinct soil types through a 101-day 4°C storage period, and found that while there were some alterations in measured functionality within soils, between-soil differences were still maintained. Likewise, Lee et al., [92] found that, for forest and agricultural soils, storage at 4°C resulted in the most similar microbial enzyme activity to the field soil, as compared to samples stored at -20°C or -80°C. They also found that the soil with the highest organic C content increased in biomass after storage, hypothesizing that the high C content supported the microbial assemblages even at the low temperature. This is similar to the increase in inferred cell abundances in our work during storage (Fig 9), and would make sense given the vast reservoir of C in thawing permafrost. However, other work has reached the opposite conclusion–for example, Stenberg et al., [93] concluded that soils frozen at -20°C were more similar to their fresh soils than soils stored at 2°C, based on their measurements of biomass, respiration, and a suite of other microbial activities. Given the variety in soil types and the variety of analyses that can be performed after storage or incubation, the effects on microbial communities during soil storage were bound to be variable.

Changes in community composition will only affect ecosystem process rates if the microbial community is (1) sensitive to the disturbance, (2) not resilient, and (3) the resulting community is functionally dissimilar to the pre-disturbance community [13,94]. In other words, for the disturbance to have a significant effect on the parameter under investigation (e.g. CO_2_:CH_4_ production rates or pathways), the disturbance must cause a lasting change in the microbial community composition such that the resulting community is not functionally similar to the original community. In their meta-analysis, Allison and Martiny [13] found evidence that microbial communities were highly sensitive and often not resilient to environmental disturbance. The microbial communities in our incubations were disturbed by collection and storage. These communities from Stordalen Mire do not appear resistant, since their compositions were altered, nor resilient, since they did not ‘bounce back’ to their original field compositions. Functional similarity is difficult to assess, given the diversity of soil microbial communities and the sheer volume of data [85]. Additionally, inferring function solely from 16S rRNA profiles can be difficult and varies depending on how phylogenetically conserved a trait is, and include the inherent caveat of potential amplification bias during PCR amplification of the 16S gene region. We therefore examined targeted metagenomes from these incubations.

#### 3.2.6 Metagenome analysis

**Fig 14:**
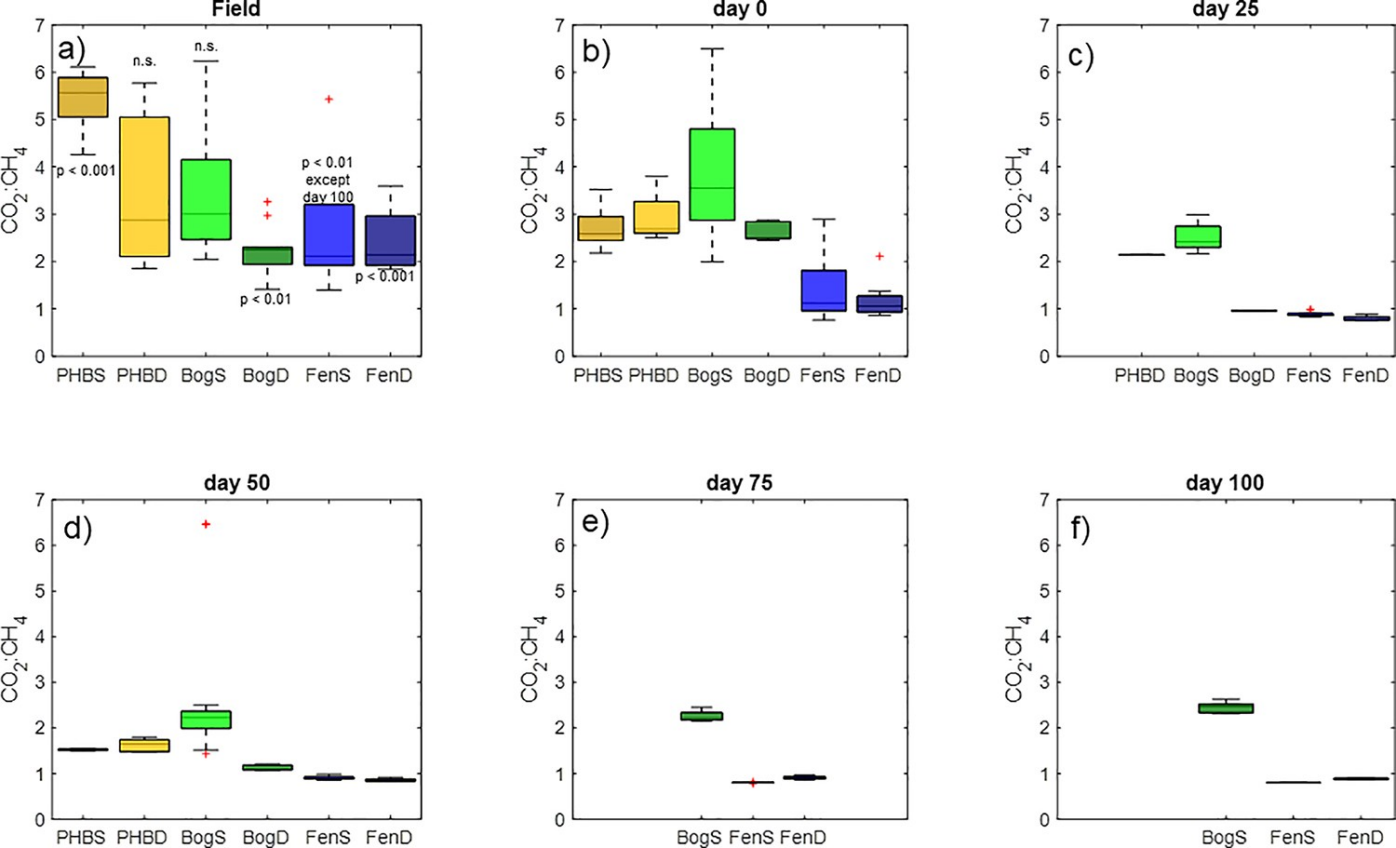
Boxplots showing CO_2_:CH_4_ ratios calculated for the field (panel a) and in the incubations averaged over 0–5 days (panel b), 20–30 days (panel c), 45–55 days (panel d), 70–80 days (panel e), and 90–100 days (panel f) throughout the incubation for each habitat. Anova was used to compare the means of each date with the field for each habitat. In panel (a) the results of that comparison are given (n.s. = p > 0.05). PHBD, BogS, and BogD did not differ from the field on any date as indicated by n.s. (i.e. not significant).

We assessed the abundance and diversity of methanogens in the field, time 0, and 50-day incubation samples for the shallow bog and fen using the methanogen marker gene *mcrA* in 6 targeted metagenomes (Fig 13). There was strong concordance in the methanogen lineages present in the field and incubation samples. The overall methanogen abundance, as inferred by the relative abundance of *mcrA*, dropped to approximately a quarter of the field by Day 0 of the incubation (after the storage and preincubation periods), and then mostly recovered by Day 50, though with more Methanobacteria. This suggests that Methanobacteria may be more sensitive to O_2_ exposure relative to the other lineages present as they decreased somewhat due to partial oxygen exposure during storage, before recovering by Day 50. In the shallow fen, the overall methanogen abundance increased over time even as the cell numbers and overall community diversity stayed flat (Figs 9 and 10), likely reflecting the fully-anaerobic nature of the incubations (with the removal of the fen’s aerenchymal gas transport to the subsurface) favoring methanogens consistent with the lower CO_2_:CH_4_ ratios observed in the incubations relative to the field (Fig 14). The portion of Methanobacteria increased appreciably, and the proportion of methanogens with the ability to consume acetate decreased, likely due to the ceased input of fresh sedge exudates, nevertheless, alpha analysis showed that the fen incubations were still strongly acetoclastic throughout the incubations (Fig 2).

## Conclusions

In the current era of high-resolution geochemistry and microbiology, incubations have the potential to clarify linkages between specific microbial lineages processes and climate-critical ecosystem outputs, and to move beyond basic representation of field carbon gas dynamics to hypothesis-testing of system responses to ongoing anthropogenic changes. For these uses of incubations to be realized, it is important to characterize the nature of the shifts in detailed geochemistry and microbiology–underlying ‘field-representative gas dynamics’—occurring within typical anaerobic incubations from climatically important wetlands.

By a number of complementary techniques, the variation in geochemical parameters appears to be driven more strongly by habitat and depth differences than by the effects of incubation. Changes over time in the incubations can be explained by expected changes to the DOM during decomposition, such as declining NOSC as labile material is used up. In many cases, the incubation CO_2_:CH_4_ ratio was not significantly different from the field. When differences were significant, the incubation ratio was lower than the field reflecting a more methanogenic (i.e. reduced) system relative to the field. For most comparisons, alphas, which indicate methanogenic pathways, were not significantly different in the field relative to the incubations. When differences were significant, the differences were slight such that the incubations correctly reflected the overall trend of hydrogenotrophic methanogenesis dominating in the collapsed palsa, a mixture of hydrogenotrophic and acetoclastic processes in the bog, and strongly acetoclastic in the fen. The preservation of this trend in the incubation samples suggest that such incubations are useful for studying environmental controls on the differences in methane production.

At various levels, examination of the microbial taxa detected in the incubations and the community structures reproduce field observations that spatial differences, by habitat and depth, were the strongest drivers of microbial community differences across the Mire. In turn, geochemical parameters were most strongly separated according to habitat types. Of the optical measurements diagenetic alteration of the material (Fig 3C), humic:fresh ratios (Fig 3D), most comparisons of humic:fulvic fractions (Fig 3E) and fulvic to fresh ratios (Fig 3F), were not significantly different at the start of the incubations relative to the field. Additionally, the high-resolution mass spectrometry data demonstrated that the incubations contained a large portion, often > 80%, of the individual DOM compounds identified in the field. Notably, the specific molecular transformations of the DOM agree well between the field and incubations (Fig 6) which together with the high percentage of field compounds captured in the incubations, suggest that the incubations are recapitulating important process-level mechanisms from the field. This reflection of field processes in the incubations occurs despite differences in the microbial community suggesting a high degree of functional redundancy in the system.

Microbial cell abundances were not significantly different from the field except in the deep bog incubations. Richness and diversity could only be significantly differentiated from the field in the shallow bog incubations. The KEGG functional analysis revealed that the majority of functional modules were similar between the field and incubation samples for a given site./depth. An examination of the microbial communities at high taxonomic resolution (OTU level) did reveal important differences between the incubations and the field that likely reflect the perturbations imposed by collection and handling of the sample, and depletion of substrates in the closed system. However, these perturbations did not seem to affect the large picture geochemistry of how decomposition occurred. Nor was their strong evidence of the build-up inhibitory compounds as evidenced by continued high production rates throughout 100 days of incubation. These results–that changes among habitats across the thaw gradient greatly exceeded variation introduced by incubation perturbations–indicate that closed-system incubations reasonably approximate conditions in the field for exploring ecosystem-scale questions about differences in CO_2_ and CH_4_ dynamics among different peatland habitat types. Therefore, incubations remain a powerful tool for understanding habitat differences at this site.

Though the microbial communities in this study were altered post-storage, with compositional differences at the OTU level, increases in richness and diversity, and temporal trends during incubation time, habitat is still the strongest driver of separation seen in these incubations. This result, suggests that the functional capacity of the communities to carry out large-scale geochemical processes is maintained during *ex situ* incubations. This is a critical finding as it suggests that despite differences in the specific microbial community, critical C cycling processes can still be conserved. This is good news for researchers wishing to scale from field observations to general global principles controlling C cycling. That differences among the different habitats was maintained in the incubations also suggests that abiotic factors have a strong control on C cycling processes. Thus, on the timescale of this study, it appears that environmental factors confer greater amounts of variation to peat decomposition than storage or ‘bottle effects’ and that such incubations remain a useful tool for studying differences in CO_2_ and CH_4_ production dynamics across the thaw gradient despite the perturbations induced by manipulation of the samples.

## Supporting information

S1 Table McrA gene abundances as determined using gene-centrically using GraftM 0.11.1 with an McrA package. (CSV)

S2 Table Biogeochemical transforms calculated for all transforms and samples. Numbers are counts of transform occurrences within each sample.
(CSV)

S3 Table Microbial cell numbers were compared relative to the field counts and the resulting p values are given in this table along with an indication of significance. (XLSX)

S4 Table Microbial diversity indices including Shannon index, Richness, and Pielous J for each sample. (CSV)

S1 Fig Heatmap of KEGG identified modules represented in the samples. (TIF)
