# Supplementary figures and images for: Functional capacities of microbial communities to carry out large scale geochemical processes are maintained during ex situ anaerobic incubation
Source: PLoS One. 2021 Feb 25;16(2):e0245857. doi: 10.1371/journal.pone.0245857 (PMC7906461; doi:10.1371/journal.pone.0245857)

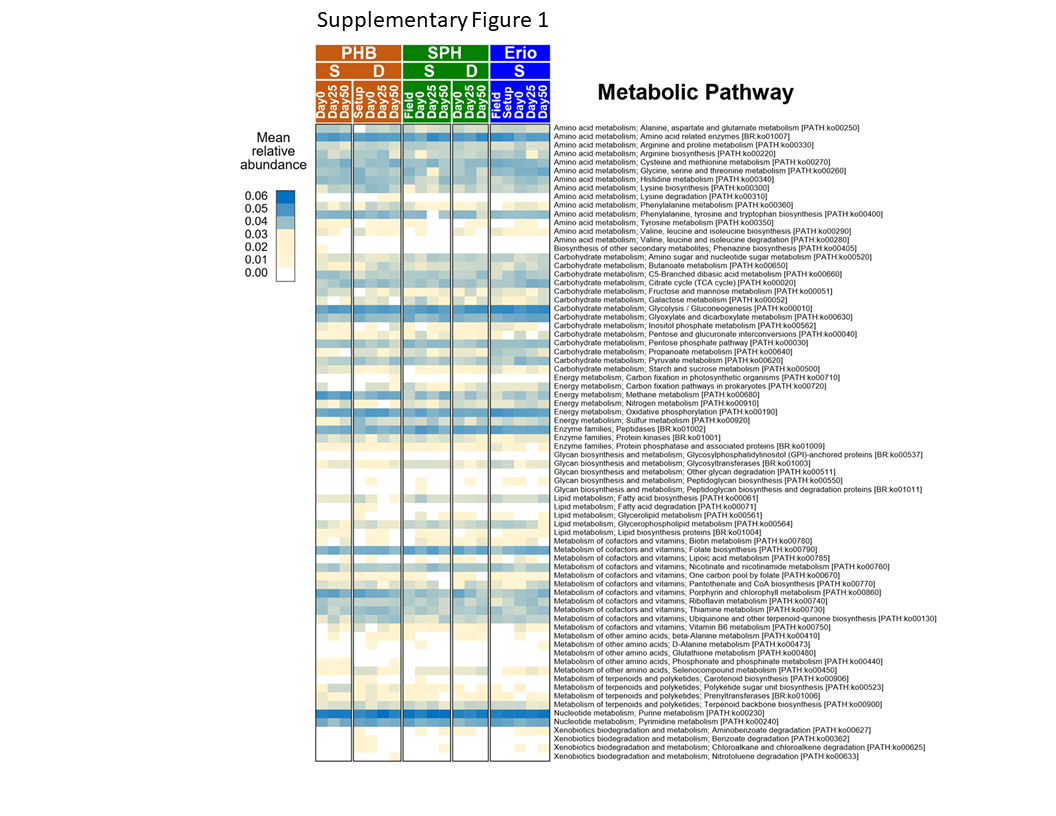

Supplement: S1 Fig — (TIF) [file pone.0245857.s005.tif]
